# Osteocyte apoptosis: the roles and key molecular mechanisms in resorption-related bone diseases

**DOI:** 10.1038/s41419-020-03059-8

**Published:** 2020-10-12

**Authors:** Jiang-ying Ru, Yan-fen Wang

**Affiliations:** 1 Department of Orthopedics, The Affiliated Hospital of Yangzhou University, Yangzhou University, Yangzhou, China; 2 Department of Pathology, The Affiliated Hospital of Yangzhou University, Yangzhou University, Yangzhou, China

**Keywords:** Apoptosis, Stress signalling

## Abstract

Vital osteocytes have been well known to function as an important orchestrator in the preservation of robustness and fidelity of the bone remodeling process. Nevertheless, some key pathological factors, such as sex steroid deficiency and excess glucocorticoids, and so on, are implicated in inducing a bulk of apoptotic osteocytes, subsequently resulting in resorption-related bone loss. As much, osteocyte apoptosis, under homeostatic conditions, is in an optimal state of balance tightly controlled by pro- and anti-apoptotic mechanism pathways. Importantly, there exist many essential signaling proteins in the process of osteocyte apoptosis, which has a crucial role in maintaining a homeostatic environment. While increasing in vitro and in vivo studies have established, in part, key signaling pathways and cross-talk mechanism on osteocyte apoptosis, intrinsic and complex mechanism underlying osteocyte apoptosis occurs in various states of pathologies remains ill-defined. In this review, we discuss not only essential pro- and anti-apoptotic signaling pathways and key biomarkers involved in these key mechanisms under different pathological agents, but also the pivotal role of apoptotic osteocytes in osteoclastogenesis-triggered bone loss, hopefully shedding new light on the attractive and proper actions of pharmacotherapeutics of targeting apoptosis and ensuing resorption-related bone diseases such as osteoporosis and fragility fractures.

## Facts

• Osteocyte apoptosis under various pathological agents has a link to resorption-associated bone fragility and bone loss.

• The morphology and vitality of osteocytes is likely an indicator of bone quality.

• A crucial cross-talk mechanism between autophagy and osteocyte apoptosis exists in maintaining osteocytic vitality.

• Key pathological agents and intrinsic signaling pathways have important roles in initiating and mediating osteocytic pro- and anti-apoptotic mechanisms.

• Apoptotic osteocytes function as a direct and indirect role in developing resorption-related bone loss.

## Open questions

• Are underlying molecular mechanisms the same whereby osteocyte cell death occurs upon various pathological agents?

• How do autophagy and osteocyte apoptosis interplay in maintaining the osteocytic vitality?

• What roles does osteocyte apoptosis have in developing the resorption-related bone loss?

## Introduction

The normal rate of osteocyte apoptosis thought as the default fate for osteocytes could result in the removal of the “dead” bone through autophagic programmed cell death, which permits the self-renewal of the bone cells and preserving of bone strength^1,2^^.^ Nevertheless, a bulk of apoptotic osteocytes are implicated in several key pathological conditions, including aging^3^, fatigue/microdamage^4^, unloading/disuse^5^, excess glucocorticoids (GCs)^6^, estrogens (Es) or androgens (As) deficiency^7^, and inflammation^8^, which are correlated with decreased bone mineral density (BMD) and increased bone loss. Indeed, targeted ablation of osteocytes was shown to induce osteoporosis with the deterioration of bone microstructure and mechanotransduction in the diphtheria toxin receptor (DTR)-9.6 kb transgenic mice, which mimicked the aging skeleton^9^. As well known, bone has a fascinating but complex hierarchical structure, whereas, osteocytes function as a pivotal role in orchestrating the preservation of bone mass and efficient load bearing^9,10^. Currently, it is widely accepted that the pulsatile fluid flow (PFF) around osteocytes may cause fluid shear stress (FSS) which affects osteocytic metabolism and vitality. Accordingly, the osteocytic gap junctions via connexin43 (Cx43) hemichannel proteins control both intercellular communication and mechanotransduction in the bone through affecting the PFF within the lacunar-canalicular network (LCN)^11^. Notably, nutrient and oxygen are transported by PFF within LCN to preserve the vitality of osteocytes, which is of paramount importance in maintaining bone mechanosensation and mechanotransduction^10,11^. Intriguingly, it was found that PFF-induced [Ca(2^+^)]i oscillations could be attenuated due to estrogen deficiency in MLO-Y4 osteocytes, which ultimately alter osteocyte function and differentiation^12^. Nevertheless, how osteocytes orchestrate the maintenance of bone mass and efficient load-bearing is not yet completely understood. Also, accumulating evidence indicates that differences in osteocyte mechanosensitivity are strictly controlled by osteocytic morphology and the lacunae size^13^. This is supported by the fact that the difference in osteocytic morphology exists in various states of pathologies. For instance, osteocytes were relatively elongated and small in the skeleton with osteoarthritis, whereas relatively round and large in osteopenic skeleton^13^. Apparently, the difference in osteocytic morphology and lacunae size would impact the cell to cell communication, subsequently reducing the structural integrity and vitality of osteocytes^14,15^. Accordingly, it is no surprise that changes to the size and density of osteocytes could be correlated with the deterioration of biomechanical property of bone tissue, consequently contributing to bone regeneration failure, as well as bone loss^16^. Specifically, irrevocable and cumulative effects, due to osteocyte apoptosis, could occur through disruption of osteocytic LCN and suppression of the repair of bone microfractures, thereby resulting in the increase of bone fragility, or maybe osteoporosis^14,15^. Based on the above reasons, the morphology of osteocytes is likely an important structural marker of osseointegration in adapting mechanoresponsivity of bone to mechanical loading, as well as an indicator of bone quality. Yet, the intrinsic mechanism underlying how osteocytic morphology affects bone architecture and function is complex and needs to be further investigated.

**Fig. 1:**
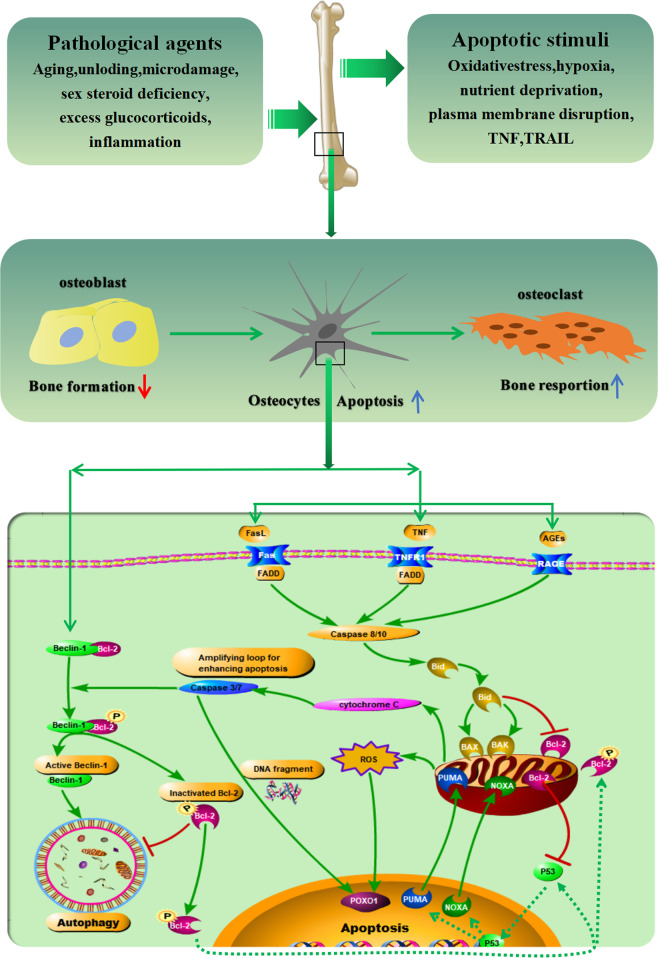
Proposed model of the interplay between osteocyte apoptosis and autophagy. Pro-apoptotic stimuli that stimulate death receptors and mitochondria-mediated apoptotic pathways give rise to the triggering of pro-survival autophagy-related biomarkers including Beclin-1 and BCL-2. In the process of continued exposure to apoptotic stimuli, phosphorylation of BCL-2, which separates from Beclin-1, translocates to mitochondria and sensitizes cells to apoptotic signals and blocks BCL-2 from suppressing pro-apoptotic biomarkers, thereby enhancing cells apoptosis. This demonstrates a mechanism of a positive-feedback loop for amplifying the death of cells.

It is intriguing that increasing experimental evidence has suggested that there exists an intrinsic cross-talk mechanism between cell death and autophagy, in which pro-apoptotic biomarkers could participate in autophagy or vice versa^17–20^. Also, it was suggested that cellular stress causes the displacement of B-cell lymphoma/leukemia 2 (BCL2) from Beclin-1 and BCL2-associated X protein (BAX), thereby triggering autophagy and apoptosis, respectively^18^ (Table 1). Based on this reason, the imbalance between apoptosis and autophagy might give rise to undesirable pathophysiological consequences. As well known, autophagy is a programmed cell survival mechanism, whereby autophagy might protect the amount of osteocyte cellular projections and retain endoplasmic reticulum and mitochondria in osteocytes^21^. Meanwhile, it is referred as a “double-edge sword” implicated in both protecting cells and enhancing the cell death^21–23^. It should be kept in mind that autophagy can be triggered by such stress factors as hypoxia, starvation, and increased oxidative stress^24,25^. Specifically, the osteocytes responded with autophagy in an attempt to “save themselves”. However, higher, or more prolonged stress could result in extensive recycling of damaged organelles and the accumulation of autophagosomes, eventually evoking cell death or apoptosis^26^. In fact, the communication of autophagy with apoptosis could be undertaken by the interplay between apoptotic proteins (e.g., cleaved-Caspase-3) and autophagic proteins (e.g., Beclin-1) (Table 1). This is manifested by the fact that the reduction of beclin-1-immunolabeled osteocytes was in parallel with the increase of cleaved-Caspase-3-immunolabeled osteocytes, thus weakening its anti-apoptotic activity^27^. In agreement with this notion, ovariectomized (OVX)-induced osteocyte apoptotic has been observed to be accompanied by a reduced Beclin-1 and microtubule-associated protein light chain 3A (MAP LC3A)-immunolabeled osteocytes and increased p62-positive osteocytes, which is abolished by the Es replacement. All these findings indicate that the intrinsic cross-talk mechanism between osteocyte autophagy and apoptosis has a crucial role in mediating cell metabolism and preserving osteocytic homeostasis^28^ (Fig. 1).

**Table 1:** Key biomarkers and their potential role in osteocytic pro- and anti-apoptotic signaling mechanisms.

Mechanism	Key biomarker	Potential roles
Pro-apoptotic function	ROS	Generate the initial insult on mitochondria; induce either apoptosis or senescence; decrease bone mass and accelerate aging; increase degeneration of the osteocyte LCN^24,25,48,72,109^
	BAX	Target the mitochondria; induce rapid release of cyt *C*; accelerate the caspase cascade^49,66^
	PUMA	Target the mitochondria; induce rapid release of cyt *C*; accelerate the caspase cascade^49^
	Hcy	Increase the expressions of Nox; induce DNA damage^96^
	HMGB1	Trigger the generation of pro-inflammatory and pro-osteoclastic factors via positive feedback loop^109–112,114^
	FADD	Trigger a caspase cascade; induce GCs-induced apoptosis^6,118^
	Sclerostin	Promote osteocyte cells death upon unloading; inhibit bone formation^53,107,108^
	BNIP3	Promote cell death during hypoxia^60,62^
	CCN2	Promote osteocyte apoptosis upon excess mechanical stress^33,69^
	TNF-α	Stimulate osteocyte apoptosis upon inflammation and cancer^92,115,116^
	Caspase-3	Regulate osteocyte apoptosis via physical interactions in mechanistic stimulus^27,127^
	CTSK	Breakdown the bone matrix adjacent to the osteocyte; increase the size of the osteocyte lacunae and mineralization decrease vitality of osteocytes^23^
	DMP-1	Regulate osteocyte formation and phosphate homeostasis; involve in osteocytic apoptosis^29,84,154^
	Pyk2	Promote GCs-induced osteocytic apoptosis via focal adhesion^82^
	Panx-1	Promote fatigue-induced osteocytic apoptosis^160^
Anti-apoptotic function	SOD2	Suppress aging and loss of bone mass; decrease degeneration of the osteocyte LCN^24,61^
	AMPK	Protects against Hcy-induced osteocyte apoptosis^96,101,102^
	NO	Maintain osteocytic vitality by pulsatile fluid flow^44,54,100^
	Cx43	Involve in gap junction; maintain intercellular communication and mechanical response^42,43,158^
	Beclin-1	Inhibit the oxidative stress; protect the survival of osteocytes^21–23,27^
	LC3	Inhibit the oxidative stress; protect the survival of osteocytes^21–23,28^
	PTH	Reverse the osteocyte apoptosis; promote gap junction-mediated intercellular coupling; stimulate Ca^+2^ influx^133–135,137^
	VEGF	Couple angiogenesis and osteogenesis; preserve osteocytic vitality^140,141^
	β1-integrin	Regulate stretch-induced ERK activation; preserve osteocytic vitality^93^
	Caveolin-1	Involve in mechanotransduction in osteocytes^55^
	PGE2	Maintain osteocytic mechanotransduction upon unloading; block GCs-induced apoptosis^56^
	PI3K	Trigger the phosphorylation and inactivate the pro-apoptotic protein; preserve osteocytic vitality^82^
	Sema3A	Inhibit osteoclastic bone resorption and promote bone formation; regulate the survival of mature osteocytes; maintain bone mass in an estrogen-dependent manner^77^
	FGF7	Increase Cx43 expression; promote gap junction elongate; maintain the survival of osteocytes^59^
	Irisin	Prevent apoptosis of osteocytes through excise^57,58,91^
	CD40	Block TNF-α or GCs-induced osteocytic apoptosis^92^
	NAC	Alleviate estrogen/androgen deficiency-induced osteocyte apoptosis^48^
	CCL7	Promote bone formation; maintain osteocytic mechanotransduction; protect from GCs-induced osteocytic apoptosis^83^
	BCL-2	Suppress osteocytic apoptosis upon unloading/disuse and fatigue^66^

All in all, despite the progress made in our understanding of the mechanism of osteocyte apoptosis and its pivotal role in resorption-related bone diseases, numerous key aspects, such as therapeutic strategies that prevent osteocyte apoptosis under pathological conditions in humans, remain enigmatic. Therefore, it might be necessary to utilize novel approaches to elucidate the findings in humans, such as the application of human-derived bone cells and immortalized bone cell lines with CRISPR-Cas9 knockout. Until recently, it has been reported that the establishment of two novel osteogenic cell lines (OmGFP66 and OmGFP10) forms 3D bone-like structures with an extensive interconnected LCN structure that mimics bone in vivo^29^. In addition, a modified method to isolate primary human osteocytes from bone also has been reported to significantly improve the yield of osteocytes^30^. The elucidation of mechanisms by more selective cells and models may have tremendous implications to inform clinical studies in humans to alleviate osteocyte apoptosis under pathologies and resorption-related bone diseases. In the present review, we summarize osteocytic pro- and anti-apoptotic mechanisms under key different pathological agents and roles of apoptotic osteocytes for a better understanding of the regulatory landscape on osteocytic life and death.

## The roles of pathological agents in osteocyte apoptosis

### Aging and senescence

**Fig. 2:**
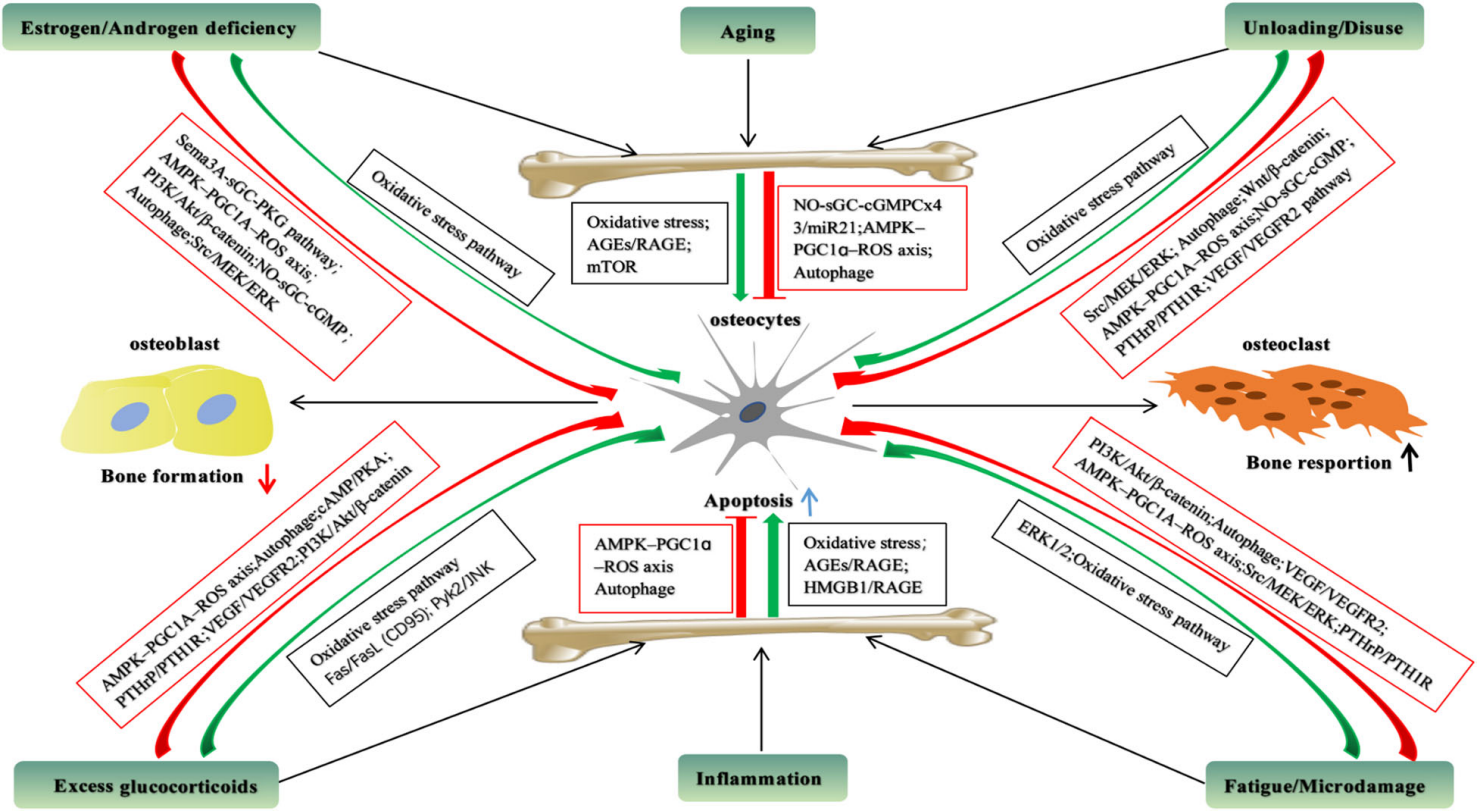
Key pro-apoptotic and anti-apoptotic pathways in osteocytes under different pathological conditions. These signaling pathways, including pro-apoptotic pathways (red arrows) and anti-apoptotic pathways (green arrows), are closely related to increased bone resorption and decreased bone formation.

Bone aging is characterized by increased “osteocyte loss”, which is associated with changes in osteocytic LCN with respect to the shape and number density^31^. Also, it was concluded that the capability to respond and repair is diminished in aged bone cells^32,33^. Morphologically, the disruption of osteocytic LCN with age decreases the provision of nutrients for osteocytes, attenuates their mechanoresponsiveness, and ultimately lead to increased bone loss and bone fragility in the elderly^14^. Indeed, the shape of osteocytes, with aging, will be smaller and more spherical. Moreover, LCN density will have a majority of the reduction in the human cancellous bone^16^, human iliac crest cortical bone^34^, and murine femoral cortical bone^35^. At large, these adverse outcomes could originate from osteocyte cell death and subsequent mineralization of osteocytic lacuna referred to as micropetrosis^16^. Recent accumulating data suggest that osteocyte death by apoptosis and the prevalence of empty lacunae are implicated age-related bone abnormalities^3,36,37^ (Table 2). It seems that the reduction of physical activity could result in, with aging, skeletal unloading to a certain extent, which gives rise to apoptosis of osteocytes^38–40^. Considering aging is commonly concomitant with increased bone loss and bone fragility, it is proposed that changes in osteocytes shape and LCN density could be correlated with an altered capability to respond to mechanical loads, which is of great importance in the preserving of bone mass and architecture^31^. Accordingly, due to aging-induced osteocyte apoptosis, the decrease in the number of osteocytes and the accumulation of mineralization in bone lacuna disturb the bone remodeling process, eventually making bone more susceptible to fragile fracture^36,37^ (Table 2). Of note, the osteocyte, one of the “permanent” cells of bone, regulates cell-to-cell communication of signals via Cx43 gap junctions to maintain cell survival, whereas, production of phosphorylated Cx43, prostaglandin E2 (PGE2), and nitric oxide (NO) would decrease with aging^41–44^ (Table 1). It should be emphasized that miR21 deletion in miR21^fl/fl^ bones increased apoptosis-related gene expression, such as phosphatase and tensin homolog (PTEN), in contrast, a miR21 analog prevented apoptosis in Cx43^def^ osteocytes, indicating that disconnection of the Cx43/miR21 pathway leads to aging-induced osteocyte apoptosis^41^ (Fig. 2). In addition, it was shown that age-related changes that occur in mitochondrial senescence or apoptosis of osteocytes comprise the reduction of mitophagy and increased uncoupling of the mitochondrion, as well as nuclear fission and fusion and increased superoxide production^45,46^^.^ Importantly, oxidative stress and resultant DNA damage (e.g., p53 and p66^Shc^), due to excessive production of reactive oxygen species (ROS), is regarded as a crucial pathway in the aging process of many cell lines including bone cells^47,48^ (Fig. 1). This is in line with the findings that AP20187, an anti-senescent drug, led to the significant reduction of both p16^Ink4a^ mRNA expression (by −59%) and enhanced green fluorescent protein (EGFP) mRNA expression (by -48%) in bone, compared with that in vehicle-treated mice, respectively, which is consistent with the clearance of both senescent cells and ensuing age-related bone loss^3^. Taken together, it is conceivable to assume that aging-induced senescence and apoptosis of osteocytes could result from the initiation of excessive oxidative stress, ensuing activation of p53, mitochondrial membrane rupture and DNA damage, as well as unleashing of cytochrome *C*^49^.

**Table 2:** Key pathological factors that are involved in the osteocyte apoptosis and their potential role.

Factor	Potential roles	Pro-apoptotic biomarkers	Anti-apoptotic biomarkers
Aging	Disruption of the Cx43/miR21 pathway; overproduction of ROS; induction of mitochondrial senescence and DNA damage	BAX, ROS, cyt C, NOXA, P53, P21	miR21, AMPK, PGC-1α, Atg7, LC3II, Beclin-1
Unloading/disuse	Generation of the senescence-associated secretory phenotype(SASP); upregulation of sclerostin to inhibit WNT/β-catenin signaling; disruption of LCN and increased localized hypoxia; overproduction of ROS	BNIP3, ROS, HIF-1α, NOX1, NOX2, Sclerostin, BAD	β1-integrin, FAK, NO, AMPK, Irisin, PGC-1α, Beclin-1, BCL-2, ORP150, PGE2, FGF7, LC3II, Cx43
Fatigue/microdamage	Rupture of dendritic processes; destroy of Cx43 gap; plasma membrane disruptions; upregulation of cellular communication network; upregulation of CCN2 through ERK1/2 pathway	HIF-1, ROS, NOX1, NOX2, Caspase-3, BAD, BAX, CCN2	FAK, c-fos, AMPK, PGC-1α, LC3II, caveolin-1, β1-integrin, Beclin-1, PGE2, Cx43, BCL-2
Estrogen/androgen deficiency	Overproduction of ROS; activation of autophagy pathway; activation of MAPK-dependent antioxidant signaling; elevation of iNOS and eNOS expression; activation of Sema3A-sGC-cGMP signalings	BAD, ROS, NOXA, P53, P66	Sema3A, AMPK, PGC-1α, NO, AKT, Beclin-1, LC3II, PI3K, RSK2
Excess glucocorticoids	Degeneration of the osteocyte LCN; upregulation of sclerostin to inhibit WNT/β-catenin signaling; activation of FAS/CD95 signalings pathway; stimulation of PTH signalings and autophagy pathway	ROS, NOX1, NOX2, CD95, Pyk2, JNK, Sclerostin, RANKL, DMP-1, CTSK, Caspases-3, -7, -8	AMPK, PGC-1α, caveolin-1, LC3II, Beclin-1, PGE2, PI3K, AKT, Cx43, caveolin-1, BCL-2
Inflammation	Unleashment of the bulk of pro-inflammatory cytokines by a positive-feedback loop; enhancement of oxidative stress; activation of AGEs/RAGE pathway	HMGB1, ROS, NOX1, NOX2, NF-κB, AP-1, CREB, STAT3, NFAT, TNF-α, IL-1β, IL-6, IL-18, VEGF-A, Caspase-1	CD40, AMPK, PGC-1α, Beclin-1, LC3II

### Unloading, nutrition deprivation, and hypoxia

As well known, osteocyte apoptosis could be decreased and bone accrual could be enhanced upon mechanical stimulation. Instead, skeletal weightlessness or unloading could result in the reverse consequence on both sides. On one hand, the data analysis suggested that apoptotic osteocytes were reduced by 40% upon short periods of mechanical stimulation, which led to peak compressive strains of 3000–4000 microstrain, compared with the unloading control^38^. It should be emphasized that FSS and muscle mass have been shown to be of great importance in the mechanical control of osteocyte apoptosis in vitro^38,50^. Similarly, the in vivo application of strains in human bone resulted in the reduction of apoptotic osteocytes, whereas unloading led to high levels of apoptosis^51^. On the other hand, due to the loss of stimuli to preserve the viability of osteocytes, unloading/disuse might contribute to more senescence and apoptosis in osteocytes. This has been manifested in spaceflight studies with rodents that help identify the pivotal role of mechanical force in maintaining osteocytic vitality. Since the effect of the decreased muscle activity during unloading or disuse, the reduction or disruption of nutrient supply could eventually result in the lipid accumulation in both osteoblasts and osteocytes^5,38^ (Table 2). Importantly, it has been assumed that the loss of mechanosensitivity, the resulting uncoupling of bone remodeling, and the generation of the senescence-associated secretory phenotype (SASP) may mainly result from osteocytic senescence and apoptosis, whether it is induced directly or indirectly during unloading^38,51^. However, it needs further research to identify the role of unloading or disuse on osteocytic senescence and apoptosis. Increasing evidence shows that osteocyte apoptosis and ensuing bone loss are both increased in both trabecular and cortical skeleton upon unloading in mice models^5,38^, which indicates that osteocyte apoptosis might have a pivotal role in regulating the adaptive response of the skeleton to changes in loading. Intriguingly, osteocyte apoptosis, during hindlimb unloading, does not occur nearer to the periosteal surface, the reason for which is likely that the fluid transport system still operates more effectively in disuse due to muscle contractions^50^. Furthermore, more apoptotic osteocytes, in disuse, occur near the endocortical surface than in the deep bone matrix, which may suggest that osteocytes near the endocortical surface are more in demand for growth factors and survival cytokines^52^. It is noteworthy that the SOST^-^ mice (the ablation of SOST gene) were resistant to disuse-induced bone loss, suggesting that sclerostin (SOST) mediates skeleton response to mechanical unloading through antagonizing Wnt/β-catenin signaling^53^. Alternatively, PFF could ameliorate disuse-induced apoptosis in osteocytic MLO-Y4 cultures, dependent on, at least partially, stimulation of nitric oxide synthase (NOS) via alterations in Caspase-3 and B-cell lymphoma-2 (BCL-2) gene expression^54^, and the involvement of mitogen-activated protein kinases (MAPKs) and initiation of the integrin/Src/extracellular-regulated protein kinases (ERK) pathway to maintain osteocytic viability^55^ (Fig. 2). Strikingly, PGE2, produced by osteocytes upon mechanical loading, may protect these osteocytes against apoptosis^56^. Nevertheless, The precise mechanism underlying how unloading/disuse induce osteocyte apoptosis is not yet completely understood. In addition, it was reported that integrins, tethering osteocytes to the canalicular wall, function as a mediator in the interaction of osteocytic processes with extracellular matrix in vivo. Hence, it has been assumed that PFF in osteocytic LCN could give rise to tension on the integrins, which in turn stimulate integrin signaling to prevent osteocyte apoptosis^55,57^. Also, it was found that the level of irisin, a peptide cleaved from Fndc5, is increased in the circulation during and following exercise in mice and humans, and its receptor, an integrin alphaV/beta 5 (αV/β5), is characterized in osteocytes^58^. Interestingly, irisin was shown to inhibit apoptosis through regulating the expression of Caspase-9 and -3 in MLO-Y4 cells and in cortical bone of osteoporotic murine models^57^. Admittedly, Cx43, as a docking platform for signal transduction, is referred to as “quality control checkpoint” in preserving the mechanosensation and mechanotransduction of osteocytic LCN^41–43^. Notably, It has been shown that fibroblast growth factor 7 (FGF7) can increase Cx43 expression and promote gap junction elongate through inducing the accumulation of cytoplasmic β-catenin and partial nuclear translocation^59^ (Table 1). As described above, the intact LCN is necessary for nutrient and oxygen exchange for osteocytes. Thus, the decrease of PFF during disuse may lead to disruption of LCN, as well as localized hypoxia in the bone. Of note, growth differentiation factor 15 (GDF15), secreted from adjacent osteocytes upon disuse and/or ischemia could function as an essential role in the promotion of osteoclastogenesis-triggered bone loss^60^. Accordingly, it is assumed that oxygen deprivation might be one main cause of the osteocyte apoptosis during unloading/disuse. This is in line with the notion that loss of mitochondrial ATP, production of reactive oxygen species (ROS), and decrease of intracellular PH (PHi) could result in cellular damage or even cell death once hypoxia and ischemia^61^. Thus, it is not surprising that mechanical stimuli are likely to promote oxygen diffusion through the movement of PFF around osteocytes, which would prevent hypoxia-induced osteocyte apoptosis. Intriguingly, a large amount of cell death may be by apoptosis, significantly precede the expression of oxygen-regulated protein 150 (ORP150) mRNA^62^, while expression of the pro-apoptotic BCL2 and adenovirus E1B19 kDa interacting protein 3 (BNIP3) is also dramatically upregulated during hypoxia^60^. It has been shown that activation of hypoxia-inducible factor-1 (HIF-1) is likely to directly stimulate the expression of BNIP3 upon hypoxia, similar to the mechanism of hypoxic induction of glycolytic enzymes (GE), vascular endothelial growth factor A (VEGF-A), and erythropoietin (EPO)^62^. It should be emphasized that ORP150 (150 kDa) deeply embedded osteocytes, a novel endoplasmic reticulum-associated chaperone^62^, has been identified to be highly expressed upon hypoxia for the formation of dendritic processes, and protection within a hypoxic environment^63^, which could have a pivotal role on protecting osteocytes from oxygen deprivation. Yet, to date, the normal scope of concentration of oxygen maintaining osteocytic vitality in LCN is still unclear. Also, the role of BNIP3 in osteocyte apoptosis needs further investigation.

### Microdamage and plasma membrane disruption

Apoptosis of osteocytes can also be induced by fatigue loading, as in the case of unloading^4,64^. The fatigue may give rise to the microdamage in the skeleton mineralized matrix, and ensuing larger bone lesions, ultimately leading to obvious fracture^4,64^. Data analysis has shown that strains of 8000 microstrains enhanced a remarkably increased osteocyte apoptosis in ulnae of rodents^65^. Similarly, it has been indicated that osteocytes will undergo apoptosis once the fatigue induces bone structural microdamage upon overloading^4,64^. All these results strongly suggest that a healthy osteocytic LCN might be of great importance in stimulating skeleton remodeling responses and/or protecting against damage, which aims to preserve bone mass and bone strength. Nevertheless, the intrinsic mechanism underlying how osteocytes die in response to microdamage or how microdamage occurs after osteocyte cell death, or both, is still ill-defined. Of interest, osteocytes are prone to give rise to apoptosis in the site close to microdamage, which is correlated with elevated expression of the pro-apoptotic biomarkers (i.e., Caspase-3, BAX). Unlike this, the expression of the anti-apoptotic protein (e.g., BCL2) is increased farther away from the damage focus^66^ (Table 1), implying that the cross talk mechanism between pro- and anti-apoptotic signals pathways has a central role in avoiding widespread cell death and maintaining bone homeostasis. In fact, the integrity of osteocytic LCN will be disrupted once microcracks occur, thus leading to the rupture of dendritic processes, which indicates that some detrimental agents including the disruption of PFF, loss of nutrients, hypoxia-induced oxidative stress, and destroy of the Cx43 gap could cooperatively give rise to osteocyte apoptosis^64^ (Table 2). Until recently, the interesting evidence indicates that tiny tears of the osteocytic membrane (from nanometers to microns), referred to as plasma membrane disruptions (PMD), are generated upon mechanical loading, which activates the osteocytic calcium signaling pathway and stimulates the upregulation of c-fos associated with the mechanoresponsivity. As a result, the ability of bone to translate mechanical stimulus into bone formation might be impaired due to loss of slower-repairing osteocytes and elevated mineralized osteocyte lacunae with aging^67^. Importantly, vitamin E, an antioxidant of interest in the musculoskeletal system, functions as an essential mediator in control of PMD-driven osteocytic adaptation and mechanoresponsivity. It has been corroborated in vitamin E-deficient diet (VEDD) mice model that the formation of PMD induced by oxidative stress was increased due to vitamin E deficiency, therefore impairing osteocyte survival^68^. In this line, the upregulation of cellular communication network factor 2 (CCN2) in osteocytes due to excessive mechanical stress is frequently addicted to osteocyte apoptosis through extracellular signal-regulated kinase 1/2 (ERK1/2) pathway via binding to integrin αVβ3^69^ (Fig. 2). Indeed, CCN2, as an essential growth factor, is well known to remarkably mediate the development of osteogenesis including endochondral ossification^69^. Based on these findings, it is conceivable that CCN2 might be upregulated in osteocytes, but not produced due to the aging-related reduction of osteocytic processes, as mechanical loading was exerted on mature osteocytes originated from the elderly mice. Consequently, apoptosis signalings might be activated since CCN2 is accumulated into mature osteocytes^69^.

### Sex steroids deficiency and oxidative stress

Es/As, as a systemic hormone, has a crucial role in the maintenance of bone remodeling homeostasis^7,70^. Indeed, osteocyte apoptosis and bone loss are frequently linked with women at menopause^70^ and animal models with Es deficiency^7,71^. In support of this notion, osteocyte apoptosis could, due to depletion of estrogen, be significantly induced in the alveolar process of OVX rodent model^28^. Likewise, because of Es deficiency, osteocyte apoptosis has been demonstrated to be dramatically increased in the long bone of OVX rodent model^48,71^. Intriguingly, apoptotic osteocytes are not located in sites of the cortex with younger osteocytes, but in sites of the cortex with oldest osteocytes. The difference of location is likely due to more sensitivity of the oldest osteocytes to the elevation of reactive oxygen species (ROS) resulted from Es deficiency^48,72^ (Table 1). In this line, in the OVX C57BL/6J rat model, it was found that increases in Casp^+^ (Caspase-positive) osteocytes were mostly positioned in the posterior cortex of diaphysis of a long bone, indicating estrogen deficiency-induced osteocyte apoptosis might be positioned locally, rather than wholely, in sites of the cortex, and apoptosis is necessary to activate endocortical remodeling following Es loss^71^. All results strongly suggest that estrogen deficiency could give rise to osteocyte apoptosis, followed by resorption-related bone loss^48,73^. It should be noted that removal of sex steroids could trigger the activation of oxidative stress in bone, including decreased antioxidant enzymes (AE) and increased phosphorylation of p53 and p66^shc^, eventually leading to accumulation of apoptotic osteocytes^72^. Consistent with this finding, oxidative stress has been shown to function as an essential pathway in regulating osteocytic viability upon Es or As deficiency via gonadectomy. Instead, oxidative stress induced by Es or As deficiency is rescued by sex steroids replacement^48^. Moreover, oxidative stress reaction and accumulation of ROS may be nullified by the administration of the antioxidant *N*-acetyl cysteine (NAC), consequently alleviating osteocyte apoptosis in gonadectomized mice^48^. Besides the antioxidant role, Es also has a pro-survival effect on osteocytes, as evidenced by the result that miR-199a-3p is involved in estrogen-mediated autophagy through the insulin-like growth factor-1 (IGF-1)/mamalian target of rapamycin (mTOR) pathway in osteocyte-like MLO-Y4 cells^73^. Indeed, autophagy could prevent estrogen deficiency-induced osteoporosis^74^. Of note, estrogen receptors (ERs), namely ERα, ERβ, and G-protein-coupled ER (GPER) have a crucial role in mediating autophagy via key signaling molecules, such as BCL2-associated athanogene 3 (BAG3)^75^. This is in line with the finding that oxidative stress induced by Es or As deficiency has been shown to be a stimuli for activation of the autophagy pathway^24^. In agreement with this notion, it is demonstrated that Es may not only activate MAPK-dependent antioxidant signaling^72^ but also the ERK1/2 pathway via binding to its receptors (e.g., ERs and ARs) to rescue osteocytes apoptosis induced by estrogen deficiency in vitro^72,74^. Importantly, the ERK1/2 pathway is one of the important pro-survival pathways that activate autophagy^76^ (Fig. 2). In addition, it was suggested that low-intensity electrical stimulation (LIES) could dramatically suppress the increased apoptotic osteocytes, and ensuing elevated empty lacunae, as well as the decreased expression of inducible nitric oxide synthase (iNOS) and endothelial nitric oxide synthase (eNOS) in OVX rats^7^. Nevertheless, it has been shown that estrogen deficiency is responded differently by the jaw, vertebral, and long bones in animal models^76^, the underlying mechanism is still not completely understood. Until recently, it was reported that osteocyte cell death, compared with littermate controls, was more significantly increased in aged Dmp1-Cre^+^ Sema3A^flox/∆^ and Dmp1-Cre^+^ Nrp1^flox/Sema-^ mice^77^. Moreover, it was found that semaphorin3A (Sema3A) or soluble guanylate cyclase (sGC) could initiate the activation of biomarkers in the cyclic guanosine monophosphate (cGMP) signaling pathway, including protein kinase G (PKG), ERKs, AKT, and vasodilator-stimulated phosphoprotein (VASP), which were suppressed by a PKG antagonist^77^^.^ Based on these findings, it was suggested that the Sema3A-Nrp1-sGC-PKG pathway functions as an essential anti-apoptotic pathway in protecting osteocytes against estrogen deficiency-induced apoptosis. Of note, Sema3A deficiency in osteocytes may result in severe osteopenia in aged mice, which could be repressed by the Sema3A-targeting miRNAs, subsequently enhancing Sema3A expression^77^ (Table 2). Here, it should be emphasized that the serum level of Sema3A could decrease with age or after menopause in humans^77^.

### GCs and autophagy

GCs are known as potent immunosuppressive and anti-inflammatory agents with the potential to inhibit the expression of several cytokines involved in inflammatory responses^6^. Thus, it is often used for the therapy of inflammatory non-infectious diseases, including rheumatoid arthritis, systemic lupus erythematosus, organ transplantation, asthma, and malignancies, etc^78–80^. Nevertheless, excessive GCs treatment could be associated with the development of over 30% of osteoporotic fracture and over 10% of osteonecrosis^6^. Importantly, it is suggested that GCs-induced bone disease is commonly characterized by the accumulation of apoptotic osteocytes in mice and humans^6,78^. In addition, the collapse of the femoral head, following GC-induced osteonecrosis, is also associated with apoptotic osteocytes localized adjacent to the subchondral region^17^. Accumulating evidence in vitro and in vivo indicates that GCs could induce osteocyte apoptosis^6,78–82^. In this line, GCs also inhibit several proteases expression, required for perilacunar skeleton remodeling, which could eventually lead to the disruption of osteocytic LCN, disorganization of collagen, and hypermineralization of the matrix, which are all found in necrotic bone lesions of human^79^. This also is supported by the fact that Wnt inhibitor genes (e.g., SOST) were expressed by osteocytes with GCs exposure, whereas concomitant autophagy mechanism also was activated, which has an anti-apoptotic role in GCs-induced osteocyte apoptosis^80–82^. Following a cumulative and unrepairable defect, bone strength could be reduced, eventually leading to the deformity of the femoral head. This is likely due to the interfering of GCs-induced apoptosis with the production of VEGF and VEGF-induced endothelial angiogenesis (Table 2). Also, tumor necrosis factor-alpha (TNF-α) and interleukin-6 (IL-6) are highly expressed in the GCs-induced apoptotic osteocytes, which in turn affects the vitality of neighboring healthy osteocytes^78^. As previously reported, it seems to exist an interplay between BCL-2 (an anti-apoptotic biomarker) and Beclin-1 (an autophagy-related biomarker), which has a crucial role in controlling the switch of autophagy and apoptosis^80^ (Fig. 1). Hence, GCs treatment could affect the expression level of the antioxidant gene in a dose-dependent manner. Specifically, GCs might activate the autophagy pathway at a lower dose level, whereas apoptosis pathways could be triggered by GCs at a higher dose level^81^. It is intriguing that the mechanism underlying GC-induced osteocyte apoptosis is also addicted to factor associated suicide (FAS)/CD95 signaling pathway, except it is mediated through the mitochondrion^6^ (Fig. 2). In addition, GCs were shown to induce apoptosis in osteocytes overexpressing wild-type proline-rich tyrosine kinase 2 (Pyk2), which was rescued in osteocytes that lacks kinase activity (K^−^; Pyk2 mutant). It was suggested that activation of Pyk2 is implicated in the process of GCs-induced osteocyte apoptosis^82^. On the contrary, mechanical stimulus-induced chemokine (C-C motif) ligand 7 (CCL7) could, in an autocrine manner, protect osteocytes from GCs-induced apoptosis selectively. Nevertheless, it was demonstrated that CCL2 expressed by MLO-Y4 cells could not only protect against osteocytic apoptosis induced by GCs, but also by TNF-α^83^. Until recently, GCs-induced apoptosis of osteocytes is shown to produce higher expression of sclerostin (SOST), receptor activator of nuclear factor-kB ligand (RANKL), and dental matrix protein 1 (DMP-1) mRNA, which could be rescued by recombinant human parathyroid hormone (rhPTH) (1–34)^84^. Importantly, DMP-1, as a crucial biomarker in mediating osteocyte homeostasis, may be required for osteocyte apoptosis^84^. Of interest, it has been found that the protective effect of rhPTH (1–34) for GCs-induced femoral head necrosis, at least in part, from enhanced autophagy due to the upregulation of LC3II and Beclin-1^84^, implying there might exist an interplay between PTH pathway and autophagy pathway. Furthermore, it is also indicated by a recent study in vivo that vitamin E, as antioxidant medication, has a crucial role in counteracting skeletal oxidative stress after excess GCs treatment, and consequently promotes osteocytic survival^85^. Nonetheless, the intrinsic cross-talk mechanism among oxidative stress, autophagy, and PTH signaling pathways in the anti-apoptotic process of osteocytes needs to be further investigated.

### TNF-α and inflammation

Currently, the burgeoning field of osteoimmunology mainly focuses on the investigation of the interaction of the immune system in the skeleton with bone physiology, of which there exist many chronic inflammatory diseases concomitant with resorption-associated bone loss, including rheumatoid arthritis (RA), spinal cord injury (SCI), and aging-related osteoporosis (OP), etc^85–89^. Though the underlying mechanism whereby some pro-inflammatory factors induce resorption-associated bone loss remains fully unknown, it is hypothesized that osteocyte apoptosis might have a profound role in regulating bone homeostasis under these chronic inflammatory diseases. It is noteworthy that osteocyte density was shown to decrease accompanied by the increased osteocyte apoptosis when the expression of TNF-α and RANKL was elevated in rats with inflammatory bowel disease^90,91^. Likewise, apoptotic osteocytes were shown to be increased in a mouse infectious osteomyelitis model, while the expression level of pro-inflammatory biomarkers was was also elevated including TNF-α, interleukin-1β (IL-1β), interleukin-17 (IL-17), and IL-6, and so on. Conversely, in the context of TNF-α deficiency mice, the inflammatory responses to osteomyelitis were significantly weakened, concomitant with fewer apoptotic osteocytes^8^ (Table 1). Accumulating evidence suggests that pro-inflammatory cytokines such as TNF-α and IL-6 could induce directly osteocyte apoptosis in cell culture models, and therefore trigger the unleashing of the bulk of pro-inflammatory cytokines by a positive-feedback loop, which is abolished by binding of CD40 ligand (CD40L) independent of signaling or transcriptional mechanisms^92^. In the line with this notion, it has been shown that apoptotic osteocytes themselves could produce pro-inflammatory biomarkers including IL-1β and interleukin-18 (IL-18)^93^ (Table 2). Intriguingly, due to the inefficient clearance of apoptotic cells, many chronic inflammatory diseases are propagated such as rheumatoid arthritis, systemic lupus erythematosus, and cystic fibrosis^89^. Based on these findings, it is tempting to speculate that apoptosis of osteocytes functions as a gatekeeper of the regulatory inflammatory responses in pathological bone diseases. Until recently, it was found that receptor for advanced glycation end products (RAGE) has an essential role in upregulating the expression level of IL-6 and VEGF-A in MLO-Y4 cells, as well as in advanced glycation end products (AGEs)-induced osteocyte apoptosis^93,94^. Specifically, it is indicated that AGEs activate the downstream signals pathways (e.g., ERK1/2, p38, and STAT3) via RAGE, thus allowing both upregulating the production of IL-6 and VEGF-A in osteocytes in a dose- and time-dependent way and inducing osteocytic apoptosis^93^ (Fig. 2). Typically, RAGE is upregulated in states of chronic inflammation, such as rheumatoid arthritis, aging- and diabetes-related osteoporosis^94^. Once AGEs bind to RAGE, the upregulation of nuclear factor kappa-B (NF-κB) is exacerbated, which in turn increases the production of AGEs, IL-6, and VEGF-A from apoptotic osteocytes in an amplifying loop mechanism, consequently promoting osteocyte apoptosis^93^. In addition, it also was suggested that not only osteocytic apoptosis but also its dysfunction both could be induced by AGEs. Of note, FOXO1, associated intensely with apoptosis, functions as a pivotal transcription biomarker in regulating the expression of Caspase-3, SOST, and RANKL, and enhancing AGEs-induced osteocytic apoptosis and dysfunction. Furthermore, FOXO1 might induce apoptosis by regulating TNF-related apoptosis-inducing ligand (TRAIL)^94^.

## The key molecular mechanisms in promoting the osteocytic apoptosis

### Oxidative stress pathway

It has been suggested that the oxidative stress pathway could be activated in a wide variety of pathophysiological agents in humans, including aging, cancer, chronic inflammatory processes^95^. Nevertheless, the precise mechanisms whereby oxidation stress signaling pathways induce cellular damage or even death remain elusive. Growing experimental evidence indicates that while oxidative stress is mediated by many oxidant and antioxidant enzymes, nicotinamide adenine dinucleotide phosphate (NADPH) oxidases (e.g., Nox1 and Nox2) are dominant oxidative stress-inductive enzymes, which could be nullified by supplementation of superoxide dismutase (SOD)^96,97^. Mitochondria is well known to be the switchboard controlling the apoptosis machinery. Once initiated, it will, as an important source of ROS, primarily targeted by the damaging effect of ROS^95^ (Fig. 1). It should be emphasized that once mitochondria are deficient in cytochrome C, ROS will be amplified to generate the initial insult, thus leading to the induction of either apoptosis or senescence since ROS accumulation^95^. Conversely, glutathione peroxidative 4 (Gpx4) has been shown to serve as a crucial phospholipid hydroperoxide glutathione peroxidase (PHGP) in defending oxidative damage of mitochondria and protecting cells from apoptosis^97^. It was reported that the levels of ROS and p16^Ink4a^, a principal effector of senescence, were upregulated in aging wild-type (WT) female and male mice. Meanwhile, it was concomitant with a reduction of glutathione reductase (GR), which is well known to protect cells from excessive oxidative stress^48,98^. Here, it should be kept in mind that p53 is implicated in cellular redox regulation as a crucial orchestrator, as well as known as a kind of SASP^98^ (Table 1). Once the activation of p53, the expression of the BCL2-associated X protein (BAX) and p53 upregulated modulator of apoptosis (PUMA) may be triggered, which targets the mitochondria to induce the rapid release of cytochrome C and subsequent acceleration of the caspase cascade^98^. It was found that increased phosphorylation of p53 and p66^shc^ activate oxidative stress pathway, therefore leading to ROS-induced osteocyte apoptosis, which indicates that oxidative stress pathway could have an important role in triggering osteocytic senescence and apoptosis upon aging^48^ (Fig. 2). In this line, a previous study by Kobayashi et al.^47^ showed that the level of ROS was significantly increased in 24 months aged mice, compared with 2 months aged mice. Intriguingly, it also be found that when osteocyte is targeted to delete the gene of mitochondrial SOD2, the levels of ROS are elevated concomitant with a low bone mass phenotype, resembling accelerated aging. Based on this finding, it is suggested that loss of SOD2 could result in degeneration of the osteocytic LCN, decreased osteocytic numbers, as well as reduced osteocytic connectivity, thereby inducing the process of osteocyte apoptosis. In addition, Piemontese et al.^99^ reported that increased bone loss and bone porosity, in the cortical skeleton of elderly C57BL/6J mice, was associated with the increased DNA damage, cellular senescence, and SASP (e.g., p53, p21), as well as the elevated expression level of RANKL in osteocytes. Thereby, it is assumed that oxidative stress evokes DNA damage, subsequently activates cellular senescence pathways, consequently leading to the development of aging-induced osteocyte apoptosis and bone loss. In support of the notion, it was shown that the age-related bone loss in mice model was rescued by suppressing the senescence of cells through novel transgenic approaches or “senolytic” drugs^3^. Besides, the oxidation stress pathway also is regarded as an essential pathway in the osteocyte apoptosis resulted from Es or As deficiency^48^. For instance, it is suggested that the production of ROS might be correlated with suppression of the NO-sGC-cGMP pathway in aging or sex steroid deficiency skeleton (Fig. 2) since excessive oxidative stress impairs the transduction of the sGC signal pathway, which is known as an essential pathway in enhancing the osteogenic effect^100^. Instead, increasing evidence has shown that 5′-AMP-activated protein kinase (AMPK)-peroxisomeproliferator-activated receptor-C coactivator-1α (PGC-1α)-ROS axis has an essential role in triggering anti-oxidative mechanisms to decrease the damage because of the unleashing of ROS. Specifically, PGC-1α has a pivotal role in regulating mitochondrial homeostasis and cell differentiation due to its ability to prevent mitochondria from ROS-controlled damage^101,102^. It should be noteworthy that osteocyte apoptosis could be induced by homocysteine (Hcy) in MLO-Y4 cells via the increased expression of Nox^97^, which was ameliorated by activation of AMPK-PGC-1α-ROS axis^96^. Until recently, it has been found that the p16^−/−^-OVX mice, compared with the WT-OVX mice, could significantly decrease the expression levels of p21 marker, indicating that p16^del^ can prevent estrogen deficiency-induced osteoporosis through suppressing the oxidative stress, osteocytic senescence, and ensuing osteoclastogenesis-triggered bone resorption. As a result, osteoblastic bone formation and osteogenesis could be enhanced^95^. All in all, the continuing investigation of how the oxidation stress pathway mediates the stimulation of osteocyte apoptosis might discover essential signaling pathways, which eventually could be applied for therapeutic intervention in resorption-associated bone diseases induced by osteocyte apoptosis.

### AGEs-RAGE/HMGB1-RAGE pathways

As described above, RAGE is expressed in many cell types including osteoblasts, osteoclasts, and osteocytes. Normally, it is, under physiological conditions, expressed in the human body at a low level, and has a crucial role in regulating bone metabolism^93,94^. Importantly, RAGE signaling is suggested to be mainly correlated with age- and inflammation-related bone diseases (Fig. 1). Once the activation of RAGE, downstream signaling proteins (e.g., NF-κB, AP-1, CREB, STAT3, NFAT) might be stimulated to induce cytokines/chemokines transcription, to control cellular processes, as well as to influence cell viability by regulating autophagy and apoptosis. However, AGEs accumulation patterns, intensities of binding RAGE/AGEs, or pro-inflammatory biomarkers components may change with aging, dependent on different organs^103^. It is well known that AGEs are irreversible Amadori products derived from protein glycation (PG), and might be present in many tissues including bone^104,105^. While AGEs can be bound to several receptors to enhance the oxidative stress reaction and effects of inflammation, RAGE is of great importance among these receptors. Once AGEs are bound to RAGE, production of ROS and pro-inflammatory cascade will be stimulated, which in turn promote AGEs formation in a positive-feedback loop^104,106^ (Fig. 2). It should be emphasized that the crosslink of proteins is implicated in the formation of AGEs. Specifically, more crosslink of matrix proteins is required for elevating the number of AGEs, which, at least in part, gives rise to deterioration of biomechanical properties in skeleton^105^. As noted, accelerated AGEs generation and accumulation are implicated in osteopenia in people with diabetes and the elderly. Instead, the diabetes-related bone loss may be rescued by irbesartan (an angiotensin II receptor antagonist) via blocking the AGEs/RAGE-mediated oxidative stress pathway^105^. Increasing evidence indicates that AGEs are crucial regulators of the pathogenesis and development of resorption-related bone diseases^93,94,106^. This is in line with the notion that Tanaka et al.^107^ reported that AGEs could enhance the production of osteocytic DNA fragments correlated with apoptosis. Also, AGEs and high glucose (HG) could give rise to deterioration of the cortical skeleton by propelling osteocyte cell death. Instead, PTH could have a positive protective role in AGEs-induced osteocyte apoptosis^107^. Similarly, a study by Notsu et al.^108^ in MLO-Y4-A2 cells has also demonstrated that osteocytic apoptosis, as well as the expression of SOST, was dramatically induced by AGE3 via the increased expression of TGF-β. However, this study did not investigate the underlying mechanisms whereby AGEs induce osteocyte apoptosis. Until recently, it is reported that the AGEs-RAGE signaling pathway is an essential pathway to induce cellular apoptosis via activating downstream proteins (e.g., ERK1/2, p38, and STAT3) in MLO-Y4 cells culture. It was found that the number of apoptotic osteocytes was more significantly elevated in the AGEs-incubated MLO-Y4 cells group (approximate 11.27%), compared with the control group (only 2.88%)^93^. Intriguingly, consistent with a previous study, AGEs were shown to promote the expression of RAGE and the unleashing of pro-inflammatory biomarkers (e.g., TNF-α, IL-6, RANKL, VEGF-A, etc) from osteocytes via a positive-feedback loop^94,107^, which might enhance the effect of AGEs-induced osteocyte apoptosis. It also is manifested by the result that when the FPS-ZM1R (an AGEs antagonist) was applied to pre-conditional osteocytes, the number of AGEs-induced apoptotic osteocytes was decreased significantly^93^. It should be noteworthy that other factors, such as ROS levels, intra- and extracellular redox conditions, and metabolic status, can also alter RAGE ligand properties and modify downstream signaling^109^, implying there might exist a cross-talk mechanism between oxidative stress pathway and RAGE pathway. In addition, high mobility group box 1 (HMGB1) has been demonstrated, as a damage-associated molecular pattern protein, to regulate essential cellular processes by interacting with two receptors including RAGE and the toll-like receptor 4 (TLR4), subsequently mediating bone homeostasis, turnover, and repair^109–112^. Importantly, HMGB1 functions as an alarmin in a warning and notifying cells about the hazardous extracellular environment in distress^111^ (Table 1). Similar to HMGB1, S100A4, bound to RAGE, also promotes the secretion of pro-inflammatory biomarkers in osteoblasts and osteocytes, and then affects the vitality of osteocytes, ultimately resulting in osteoclastogenesis-triggered bone resorption^113^. Some preliminary evidence suggests that HMGB1, thought as a pro-inflammatory cytokine, might be released by senescent or apoptotic osteocytes, thereby stimulating the generation of pro-inflammatory cytokines (e.g., TNF-α, IL-6, RANKL, etc) by activating RAGE from osteoclasts and osteocytes^111,112,114^. Accumulating evidence suggests that pro-inflammatory cytokines (e.g., TNF-α, IL-1β, etc) directly induce osteocyte apoptosis in cell culture models, therefore triggering the release of cytokines that influence bone turnover^92,111,115^. Also, TNF-α binds to its receptor, TNF receptor 1(TNFR1), triggering Caspase-8 activation, and ensuing Caspase-3 stimulation, which results in the induction of apoptosis^116^ (Fig. 1). In addition, the formation of a multinuclear actin ring induced by HMGB1 was suppressed in RAGE^−/−^ pre-osteoclasts, implying there might exist an interplay between HMGB1 and RAGE^112^. Therefore, it is assumed that HMGB1-RAGE signaling pathways might be a crucial pathway in enhancing osteocyte apoptosis in the inflammation-related bone diseases, which, nevertheless, needs to be further corroborated by more studies (Table 2).

### FAS/FASL pathway

The FAS/FAS ligand (FASL) pathway is well thought as an essential mechanism in removing misplaced and/or displaced cells in immune tissues or organs^6^. It should be kept in mind that FASL is one of the TNF family and is mainly synthesized as a membrane protein (type II). Specifically, the cytoplasm contains N terminus of FASL, whereas, the region of C terminus, in common, is stretched out to the extracellular space^117^. When the FAS receptor (FASR) is bound to FASL, it will oligomerize and recruit the FAS-associated protein with a novel death domain (DD), then interact with Caspase-8, and, in consequence, trigger a caspase cascade leading to apoptosis^6^ (Fig. 1). It has been suggested that FAS/FASL apoptotic pathway is implicated in GCs-induced apoptosis in several different cell types^6,117^ (Fig. 2). Nevertheless, whether bone homeostasis is controlled by FAS-mediated apoptosis is still poorly understood. It is noteworthy that the FASL deficiency mice (FASL^CKO^) showed an osteopenic phenotype more remarkably, compared with the littermate control (FASL^fl/fl^). Meanwhile, the number of apoptotic osteoclasts (TUNEL^+^; TRAP^+^) was significantly decreased, indicating that the enhanced osteoclastic activity is most likely due to the reduction of apoptotic osteoclasts^118^. Importantly, because of the detection of apoptosis-associated biomarkers (e.g., BCL-2, FAS, BAX, and FASL, etc) and cells apoptosis, it is assumed that part of osteoblasts and osteocytes could give rise to apoptosis when osteoblasts differentiate to form osteocytes early, which is tightly controlled by the appropriate number of mature osteocytes in the bone to maintain the optimal condition^118^. Similarly, the receptor of FAS/CD95 mRNA was found to be expressed in MLO-Y4 cells whether these osteocytes were pre-treated by GCs or not^117^. In addition, it was shown that GCs could, in a Caspase-8-dependent pattern, induce osteocyte apoptosis via the FAS/CD95 death receptor^6^, which is inhibited by all variants of the bisphosphonates (BPs) molecules including non-N-BPs and N-BPs^6^. It is intriguing that the upregulation of FAS was found to not be accompanied by high expression of FASL, pointing to the possible existence of FAS-mediated osteocyte apoptosis independent of FASL^117^.

## The key molecular mechanisms in preserving the osteocytic vitality

### Autophagy pathway

**Fig. 3:**
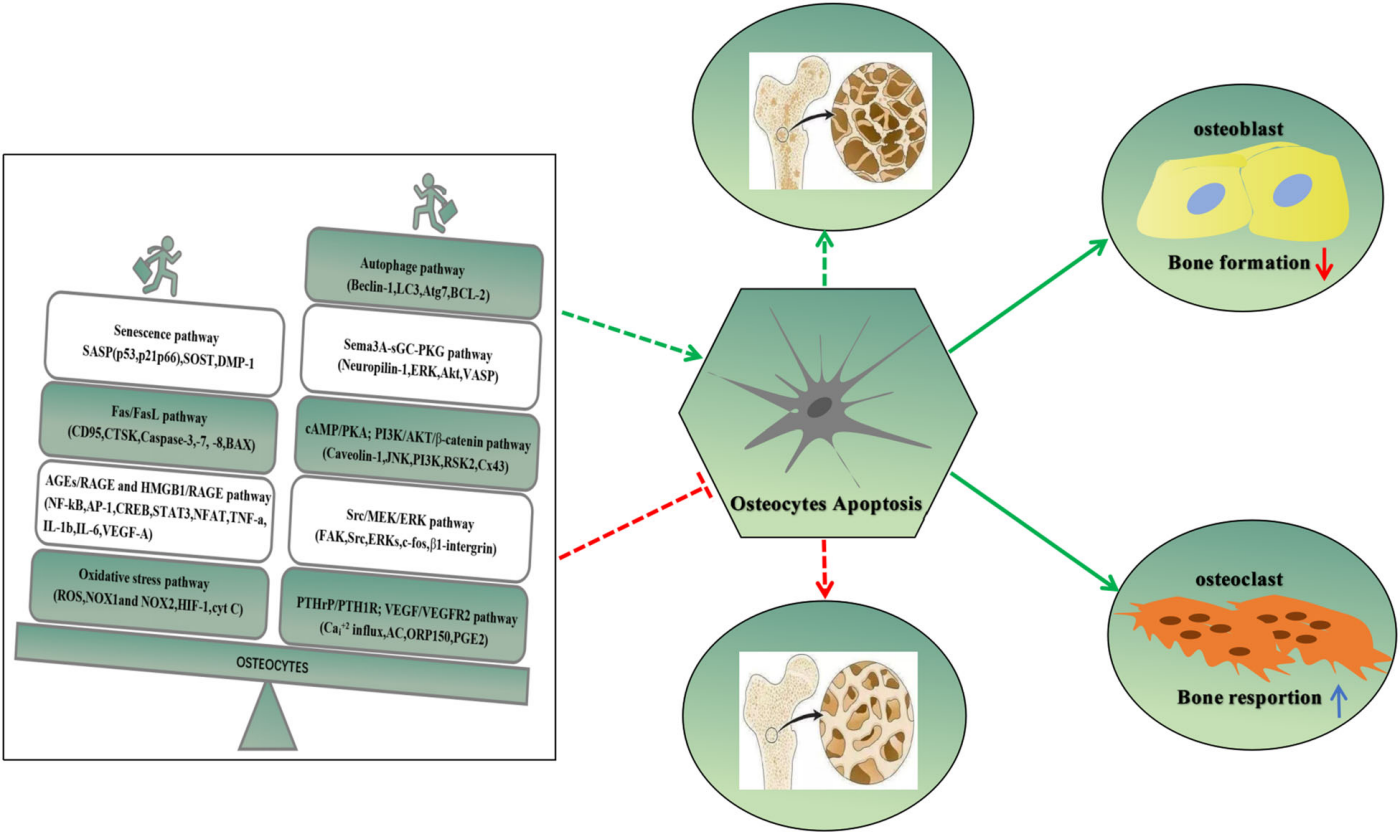
The balance between pro- and anti-apoptotic pathways of osteocyte apoptosis. Under physiological conditions, it is in an optimal state of balance tightly controlled by pro- and anti-apoptotic mechanism pathways. Once this balance is upset, masses of osteocytes may undergo apoptosis, thereby resulting in bone metabolism disorder.

Autophagy is a well-conserved mechanism among species and is involved in various biological events^20^^.^ Specifically, autophagy, as a “self-devouring” autophagosome-lysosomal process, is triggered to adapt to the stressful environment to preserve the vitality and function of long-lived cells by degrading and recycling damaged organelles and macromolecules^21^^.^ Importantly, starvation, referred to as a crucial stimulus of autophagy, could promote the generation of metabolites through degrading the intracellular constituents to preserve cell vitality, which is required for fueling the respiration of mitochondria and production of ATP^119^^.^ Not surprisingly, this pathway should also be implicated in osteocyte metabolism. Currently, several specific autophagy-related receptors and key biomarkers, known as crucial mediators in the selective degradation of pathogens, dysfunctional organelles, and aggregated proteins, have been screened and characterized partly. However, how selective autophagy has a physiological role is not yet completely understood^120^^.^ Of note in this regard, the punctuate distribution of MAP1LC3A was both shown in osteocytes of the murine and human cortical skeleton, indicative of autophagy. In addition, autophagy flux, in an in vivo study, was upregulated when osteocytes encounter stress conditions such as deprivation of nutrition and hypoxia^21^^.^ Therefore, it is suggested that the elevation of autophagic flux is required for the survival of differentiated osteocytes in stressful conditions. Accumulated evidence indicates that the autophagy pathway has a protective role in preserving the osteocytic vitality under the state of low oxygen pressure (PO2^) or^ low-dose GCs therapy^80,81,121^. In this line, it was demonstrated that autophagy, as an anti-apoptotic factor, could be negatively mediated via the mTOR pathway with aging, thereby leading to cell senescence and apoptosis^122^. Likewise, bone turnover was reduced and p66^shc^ phosphorylation was increased in L6 vertebrae of Dmp1-Cre; Atg7f/f mice, suggesting inhibition of osteocytic autophagy could generate the evidence of bone aging in many ways^123^. Consistent with this findings, it was suggested that the secretion of LC3II/I, Unc-51 like kinase 1 (Ulk-1), and Beclin-1 was significantly reduced with aging, while the expression of SQSTM1/p62 (sequestosome 1) and osteocytic apoptosis were dramatically elevated, implying senile osteoporosis concomitant with bone loss might be attributed to weakening osteocytic autophagy, which is independent of apoptosis^124^ (Table 1). This also is in line with the result that autophagy might protect the skeleton against age-related bone loss, in part at least, through decreasing the activity of apoptosis, whereas, suppression of autophagy could result in the elevation of damaged mitochondria and oxidative stress product (e.g., ROS)^121–123,125^ (Table 1). It is intriguing that Beclin-1, an anti-apoptotic biomarker in autophagy, could be cleaved by caspases, thereby acquiring a new function and amplifying mitochondrion-mediated apoptosis due to the C-terminal fragment of Beclin-1^126,127^, implying a cross-talk mechanism between autophagy and apoptosis (Fig. 1). Indeed, osteocyte apoptosis, under physiological conditions, is in an optimal state of balance tightly controlled by pro- and anti-apoptotic mechanism pathways. Once this balance is upset, masses of osteocytes may undergo apoptosis (Fig. 3). In this line, it was reported that GCs-induced apoptosis was increased in MLO-Y4 osteocytes in a dose- and time-dependent pattern. Meanwhile, autophagy markers (LC3II and Beclin-1) were increased at the low dose of dexamethasone (10^−7^ or 10^−6^ M), and yet decreased at the high dose (10^−5^ M), indicating that the self-activation of autophagy may be a protective mechanism against GCs-induced apoptosis^84^.

Nevertheless, there are controversial observations, including (1) no significant relationship between autophagy and apoptosis in osteocytes during aging^124^ and (2) no any changes in the number of osteocytes or osteocytic apoptosis in the transgenetic mice (i.e., Atg7^del^ by the Dmp1^-Cre^ transgene)^123^. It should be emphasized that if autophagy can never become inactive until osteoblasts differentiate into the stage of matrix-synthesizing, thus it is not impossible to allow autophagy to continue until osteocytes were fully formed^123^. Accordingly, it is no surprise that the formation of osteocytes and maintenance of vitality may be influenced if autophagy becomes inactive at earlier stages of osteoblast differentiation. Based on above reasons, the controversial problems in these studies could be, at least in part, explained. Nonetheless, the molecular mechanisms about potential effect of autophagy on osteocytic apoptosis need to be further established.

### PTHrP/PTH1R and VEGF/VEGFR2 pathways

**Fig. 4:**
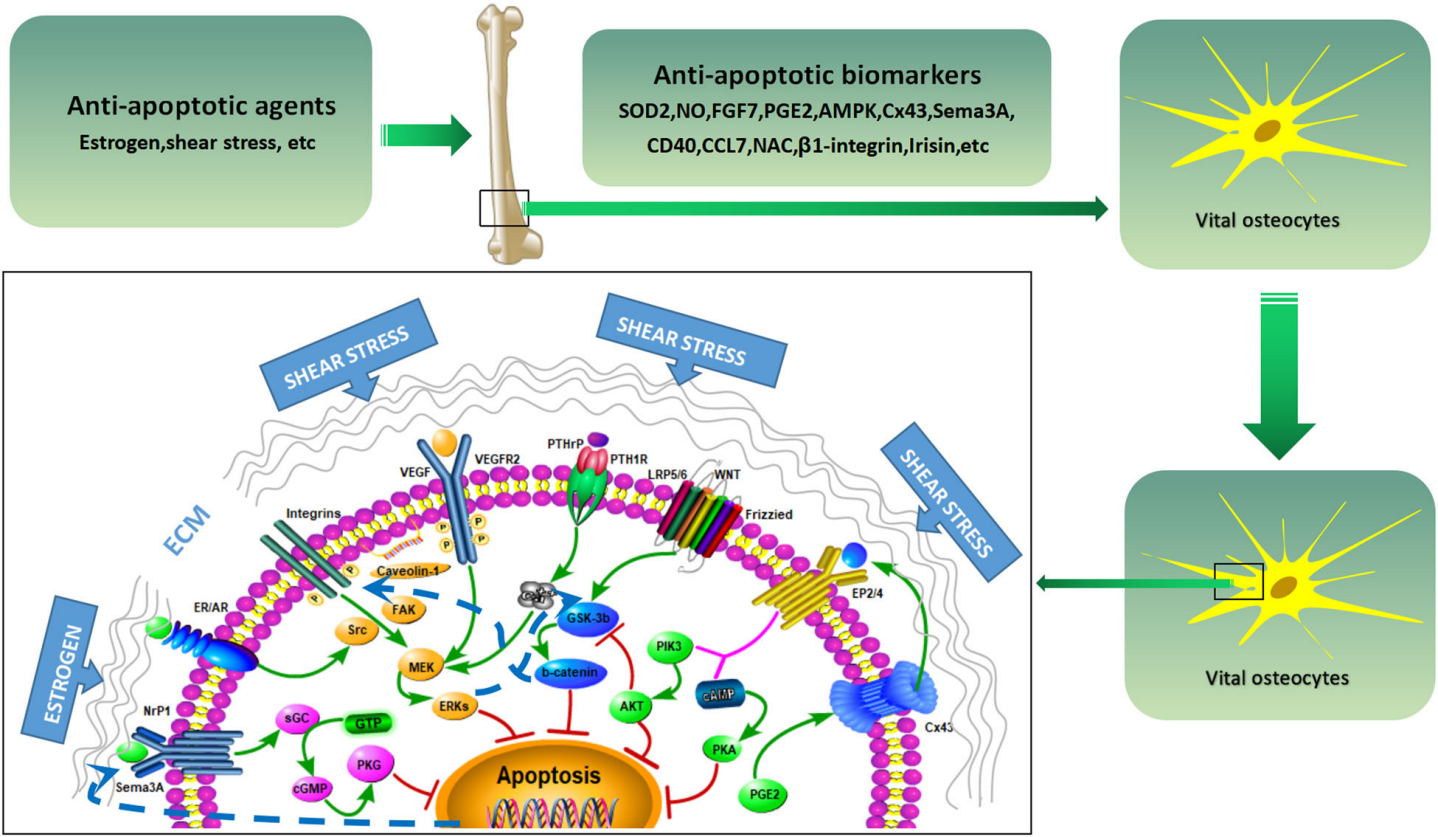
Model of anti-apoptotic signaling pathways in osteocytes. Initiation of the mitogen-activated protein kinase (MAPK) cascade is triggered by key pathological agents including Es, FSS, and so on, thereby promoting cell survival. Besides, the cAMP/PKA pathway, Sema3A-Nrp1-sGC-cGMP pathway, PTHrP/PTH1R system, and VEGF/VEGFR2 system activation also functions as a cooperator during this process. Intriguingly, there exists a cross-talk mechanism among caveolin-1/ERKs signalings and Wnt/β-catenin signalings and PI3k/AKT signalings.

As well known, osteocytes could express parathyroid hormone-related protein (PTHrP) type 1 receptors (PTH1R), which are fully activated by PTH fragments and coupling to multiple signal transducers, including adenylyl cyclase(AC) and phospholipase C^128,129^. It has been found that osteocyte-selective PTH/PTHrP receptors (PPR) knockout (Ocy-PPR^cKO^ mice) revealed a reduction in trabecular bone and slight osteopenia correlated with the elevated expression of SOST, which, yet, were unable to be suppressed by PTH in the Ocy-PPR^cKO^ animals^128^. Accordingly, it is suggested that PTH functions as an important protective role in mediating the skeleton remodeling and calcium homeostasis. Of note, intermittent parathyroid hormone (iPTH) is highly correlated with its anti-apoptotic effect on both osteoblasts and osteocytes, however, production of excessive PTH following primary hyperparathyroidism or calcium deficiency could give rise to bone loss, which is attributed to the upregulation of fibroblast growth factor 23 (FGF23), and suppression of osteoblast differentiation and mineralization^78,130,131^. It should be emphasized that SOST-positive osteocytes of the cortical skeleton in Dmp1Cre.Pthlh^f/f^ mice models were more significantly than that in Dmp1Cre.Pthlh^w/w^ controls, and decreased bone mass and increased bone resorption were shown in Dmp1Cre.Pthlh^f/f^ mice, indicating the full length of PTHrP derived from osteocytes stimulate skeleton formation and enhances skeleton strength in an autocrine and paracrine manner, in which actions might either be mediated by PTH1R or independent of PTH1R^129^. Pharmacologically, it was shown that application of iPTH (1–34) could remodel and strengthen skeleton structure in a time- and dose-dependent pattern, which was especially obvious in the trabecular region of the long bone in mice model^131^, as evidenced by the findings that rhPTH (1–34) could reverse GCs-induced osteocyte apoptosis not only in cell numbers but also in the function^84^. In this line, it was found that deletion of PTH1R in transgenic mice displayed slight osteopenia and disruption of calcium homeostasis, indicating that PTH/PTH1R pathway has a crucial role in preserving bone mass^128^ (Fig. 2). Importantly, PTHrP, much like PTH, displays anti-apoptotic features in osteoblasts and osteocytes^132–134^. Similarly, it also was corroborated that many of the osteocyte-related genes, such as SOST, DMP-1, were downregulated by PTH in an expected manner in OmGFP66 cells (immortalized osteogenic cell lines) derived from primary osteocytes of transgenic mice (DMP1-promoter)^29^. Based on these findings, it is demonstrated that the PTHrP/PTH1R pathway functions as an essential pathway in preserving the osteocytic vitality upon mechanical stimulus (Fig. 3). Nevertheless, the mechanism whereby PTH prevents osteocyte apoptosis remains ill-defined. As noted, it was reported that activation of PTH1R in osteocytes could promote gap junction-mediated intercellular coupling, increase expression of MMP-9, and potentiate calcium influx via stretch-activated Ca_i_^2+^ channels, as well as amply the osteogenic response to mechanical loading in vivo, therefore leading to protect osteocytes from apoptosis^135^. Consistent with this notion, it is suggested that G-protein coupled receptors (GPCR) may participate in the cell transduction of mechanical signals, PTH1R and stretch-activated Ca_i_^2+^ channels might both be part of the same mechanosensor system^136^. Of note, both Ca_i_^2+^ and cAMP-dependent pathways activated by upregulation of PTHrP, as exhibited in an in vitro study, could have a cooperative role in maintaining MLO-Y4 cell viability^133^. Different from these results, it has been demonstrated that the expression level of SOST is decreased and Wnt/β-catenin signalings are activated in the transgenic mice (DMP1-caPTH1R), whereas, while these mice were crossed with mice lacking the low-density lipoprotein (LDL) related receptor 5 (LRP5) (Wnt co-receptor, LRP5^−/−^) or with mice overexpressing SOST (the Wnt antagonist) (DMP1-SOST), the skeleton phenotype is, in part, reversed in the DMP1-caPTHR1; LRP5^−/−^ mice and abolished in DMP1- caPTHR1; DMP1-SOST mice, indicating that PTH1R from osteocytes has a pivotal role in promoting bone formation via Wnt/β-catenin signaling^137^. Strikingly, the inactivation of the pro-apoptotic protein, such as BCL2-associated death promoter (BAD), and increased synthesis of the anti- apoptotic protein (e.g., BCL2) are required for the PTH-induced cell survival (Table 1). It should be emphasized that Cx43 mutants (Cx43^∆245^ or Cx43^S368A^), thought as disruption of Cx43 communication and function, was shown to fail to recover PTH-induced osteoblast survival in Cx43 deficiency bone cells. Likewise, over-expression or phosphorylation of Cx43 in wild-type mice, but not Cx43^S368A^ or Cx43^∆245^, also decreased the interplay between PTH1R and β-arrestin^138^, implying interaction Cx43 with β-arrestin is a prerequisite for osteoblast survival induced by PTH. It is intriguing that whether PTH produces an anti-apoptotic or pro-apoptotic effect is dependent on cAMP activation in the cAMP signaling pathway, which has a critical role in controlling autophagy via ERK-mediated induction of cyclin E and recruitment of Beclin-1^84,139^ (Fig. 4). Besides the PTHrP/PTH1R system, VEGF/VEGF receptor2(VEGFR2) system is well known as an important mediator in proliferation, differentiation, and survival of bone cells, including osteoblasts, osteoclasts, and osteocytes^140^. Indeed, VEGF, as a major angiogenic factor, has a crucial role in coupling angiogenesis and osteogenesis via binding to VEGFR2^140,141^. It is noteworthy that VEGF, secreted by neighboring healthy osteocytes, was shown to be decreased in apoptotic osteocytes, and to be increased in sites far away from microdamage, suggesting that VEGF from osteocytes could have a pro-survival effect on osteocytes^141^. In agreement with this notion, it has been demonstrated that mechanical stimulus and VEGF both stimulated the secretion of anti-apoptotic biomarker BCL2, whereas, pro-apoptotic biomarker BAX was suppressed via ERK signalings^140^ (Table 1). Strikingly, different from non-transfected or Scr siRNA-transfected MLO-Y4 cells, PFF-induced VEGFR2 phosphorylation, via both Tyr-1059 and Tyr-1175, was dramatically impaired in caveolin-1 siRNA-transfected MLO-Y4 cells, which is correlated with re-localization of β-catenin into the osteocytic membrane^140^ (Fig. 4). Hence, it is supposed that VEGFR2/caveolin-1 might be an essential pathway, as parts of the signalosomes, in the anti-apoptotic osteocyte protection associated with mechanical loading^140^.

### Src/MEK/ERK pathway

It has been demonstrated in an in vitro study that the stretching of MLO-Y4 cells stimulated an anti-apoptotic effect via a complex signaling pathway including such signalosomes as integrins, Src kinases, and extracellular signal-regulated kinases (ERKs)^55^. It has been suggested that mechanical stimuli promote osteocyte viability via activation of Src/MEK/ERK signal pathway (Fig. 2). Importantly, these signalsomes are involved in the activation of Src/MEK/ERK signal pathway including integrins, actins, microtubular cytoskeletons, FAK, Src kinase, and Shc, which cooperatively transduct mechanical signals into ERK activation^69^. Specifically, Src is required not only for phosphorylation of substrates (e.g., Shc), but also for assembling of the signalosomes, which makes it possible to transduct the stretching stimulus signals via the interplay of protein SH2 domain, indicating the SH2 domain of Src and the kinases are both necessary for stretching stimulus to have an anti-apoptotic role^74^. Consistent with the fact that there exists the high expression level of β1-integrin in MLO-Y4 osteocytic cells, but not β2-integrin, it was demonstrated that β1-integrin, ɑ2-integrin, and ɑ5-integrin stimulated the initiation of ERK pathway induced by stretching through activating intracellular anti-apoptotic biomarkers^93^. It should be emphasized that activation of ERK and mechanical stimulus-induced osteocytic survival were reversed by β-cyclodextrin, which disrupted the microdomains of the cell membranes as a cholesterol chelator^74^. Of note, caveolin-1, known as an important structural constituent of caveolae, could interact with β1-integrin and ERKs in MLO-Y4 cells^55^, indicating caveolin-1 is involved in transduction of mechanical signals in osteocytes. Indeed, sex steroids (e.g., Es, As) also eliminate the osteocyte cell death through promptly triggering the Src/ERK pathway^142^ via ERs α/β and ARs^70^ (Fig. 4). Whereas, BPs or alendronates (ALNs) functions as anti-apoptotic drugs by activating ERKs signaling pathway and opening Cx43 hemichannels^138^. Nonetheless, how ERK pathway is exactly activated and how ERKs stimulate the osteocytic survival both remain ill-defined. As noted, the function of Cx43 hemichannels are correlated with the activation of kinase Src in the Src/MEK/ERK signal pathway, in which the binding sites of SH2 and SH3, domains of Src, are involved^138^. Specifically, the pore formation of C-terminal domain of Cx43, activation of Src kinase, stimulation of kinase ERK, and the binding actions of SH2 and SH3 are required for the transduction of mechanical signals via Src/MEK/ERK signal pathway^41,42^. It is intriguing that once Cx43 hemichannels are phosphorylated by Src or ERKs, its closure could be induced by a feedback loop mechanism^41,138^. In this situation, activation of Src could not only stimulate signals of cell survival via activation of ERKs, but also turn off Cx43 hemichannels to maintain the balance of cell homeostasis^42^. As described above, it is shown that Cx43 hemichannels are necessary for BPs or ALNs to have the anti-apoptotic effects on osteocytes^41,138^. Earlier in vitro studies has corroborated that the opening of Cx43 hemichannels is involved in the mechanism whereby BPs or ALNs generate the anti-apoptotic effect on osteocytes via triggering the Src/MEK/ERK pathway^42,138^. Of interest, unlike most agents that induce ERKs activation, BPs could activate ERKs, independent of the mevalonate pathway, to promote the osteocytic survival, which is mimicked by IG9402 (a bisphosphonate analog) independent of osteoclasts^143^.

### cAMP/PKA and PI3K/AKT/β-catenin pathways

As described above, FSS might share the same pathways with GCs in the process of osteocyte apoptosis. It has been demonstrated that FSS could suppress GCs-induced apoptosis in MLO-Y4 osteocyte-like cells via the cross-talk mechanism between β-catenin pathway and the AKT/phosphatidylinositol 3-kinase (PI3K) pathway, as well as the classic protein kinase A (PKA)/cAMP pathway, in which PGE2 has an essential role of mediator^56^ (Fig. 4). Indeed, PGE2, as a factor maintaining osteocytic vitality, is shown to be produced by osteocytes in response to mechanical loading. Notably, the anti-apoptotic role of PGE2 is controlled by binding to EP2 and EP4 receptors, and ensuing activating cAMP/PKA pathway in the protection of osteocytic vitality upon FSS^56^. Intriguingly, not only the cAMP/PKA pathway but also β-catenin signaling pathways is implicated in protecting osteocyte against apoptosis by FSS. It has been suggested that there exists a cross-talk mechanism between these two pathways^56^. In agreement with the notion, it also has been indicated that sex steroids-activated PI3K might trigger the phosphorylation of the pro-apoptotic biomarker BAD and ensuing its inactivation, which is most likely due to an action of ribosomal S6 kinase 2 (Rsk2), apart from the anti-apoptotic effect via ERKs/JNK-triggered signalings pathway^142^. In addition, it was found that mechanical stimulus resulting in osteocytic survival is tightly mediated by bidirectional cross-talk mechanism between Wnt/β-catenin signalings pathway and ERKs pathway (Fig. 3), as evidenced by the result that blockade of the caveolin-1/ERK pathway obliterates phosphorylation of glucogen synthase kinase 3 (GSK3) and accumulation of β-catenin induced by mechanical stimulation^144^. Until recently, it was revealed that exosomes inhibited GCs-induced osteocyte apoptosis through activating miR21-PTEN-AKT signaling pathway^145^. Based on these results, it is proposed that β-catenin accumulation is required for osteocytes to maintain the mechanical properties. Of course, localization of β-catenin within caveolae is also essential for osteocytes to fully activate the ERKs pathway and promote the survival of osteocytes. Meanwhile, mechanical stimulus-triggered ERK activation, in turn, is also a precondition for accumulation of β-catenin by a bidirectional regulation loop^145^. Nonetheless, the complex cross-talk mechanism among PI3K/AKT pathway, β-catenin signaling, cAMP/PKA pathway, and ERKs signalings need to be further investigated.

### Sema3A-Nrp1-sGC-PKG pathway

Sema3A, as a potent axon guidance molecule, is known to increase skeleton mass by suppression of skeleton resorption and enhancement of skeleton formation, suggesting that Sema3A also functions as a crucial osteoprotective role^146^. However, it needs the further study to explicit what the relationship between Sema3A and the neural system is. Until recently, the evidence indicates that Sema3A, derived from osteoblast lineage cells and independent of neural system, has a fundamental role in regulating the interaction of bone cells including osteoclasts, osteoblasts, and osteocytes, as well as controlling bone mass in a paracrine and autocrine manner in the postnatal period^77^. Also, it is clearly demonstrated in aged mice model that osteocytes are a major source of Sema3A, which specifically regulates the survival of mature osteocytes, as evidenced by the result that the survival of osteocytic IDG-SW3 cells, but not the survival of undifferentiated cell, was enhanced by treatment with Sema3A^77^. Of note in this regard, polymorphisms in PLXNA2, which encodes Plexin-A2 and participates in a receptor complex for Sema3A together with neuropilin-1 (Nrp1), are correlated with lower bone mineral density (BMD) and higher fracture risk in post-menopausal women^147^, indicating Sema3A serves as a crucial anti-apoptotic factor in maintaining the osteocytic vitality and skeleton strength upon Es (Table 2). Consistent with this evidence, it was found that Es could inhibit the secretion of microRNAs (miR-497 and miR-195), which acts on untranslated region (UTR) of Sema3A gene to reduce the production of Sema3A, therefore elevating the expression level of Sema3A^77^. As previously reported, Gucy1a1 is well known to encode the subunit of soluble guanylate cyclase (sGC), and to be only secreted in IDG-SW3 osteocytes and primary osteocyte-enriched population. Importantly, cGMP/NO-stimulated initiation of AKT and AKT/cGMP/PKG-dependent activation of BAD is implicated in estrogen-mediated pro-survival of osteocytes^148^^.^ Likewise, Sema3A, as a member of semaphorin family proteins, activates sGC-cGMP signaling to protect osteocytes from apoptosis (Fig. 4), and consequently ameliorates bone loss after OVX^146^. Specifically, when Sema3A or sGC activator in the sGC-PKG pathway is triggered and downstream signalosomes including AKT, ERKs, and VASP are activated, the pro-apoptotic protein cleaved-Caspase-3 was diminished but restored by the inhibition of PKG^77^. Taken together, Sema3A-sGC-cGMP signaling might be shared in mediating bone homeostasis upon Es deficiency and aging, thereby providing a new approach for the therapy of resorption-related bone disease through targeting Sema3A to promote the survival of osteocytes.

## The role of apoptotic osteocytes in osteoclastogenesis-triggered bone loss

**Fig. 5:**
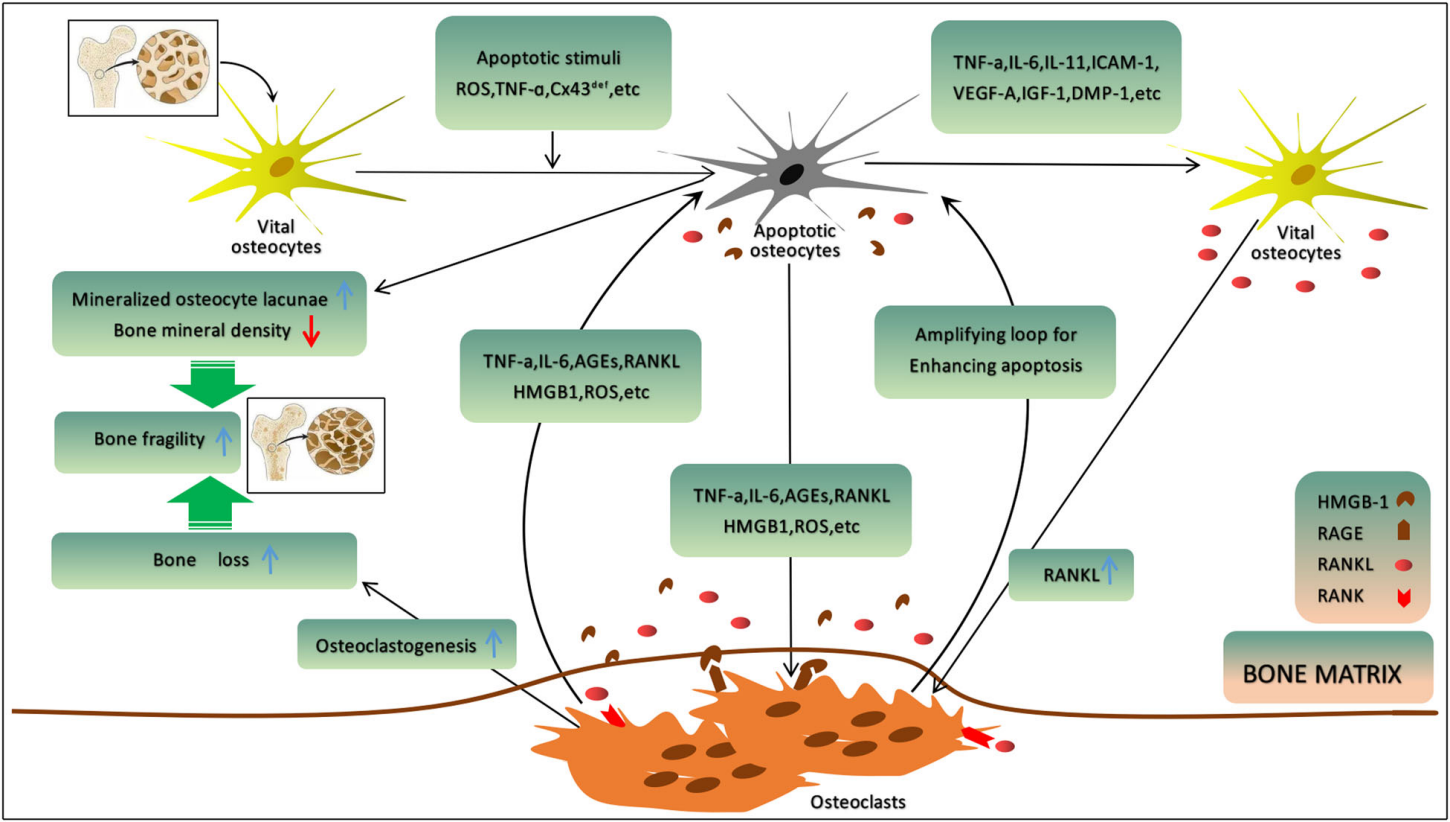
Model of the role of osteocyte apoptosis in osteoclastogenesis-triggered bone loss. The direct and indirect role of osteocyte apoptosis in osteoclastogenesis, eventually, results in increased bone loss and bone fragility. During sustained exposure to apoptotic stimuli, masses of osteocytes may undergo apoptosis, concomitant with the unleashing of the pro-inflammatory and pro-osteoclastogenic biomarkers from osteocytes and osteoclasts cytokines including RANKL, HMGB1, TNF-ɑ, IL-6, etc, which in turn promote osteocytic apoptosis by an amplifying loop mechanism.

To date, many studies have been addressing the question whether there is a causative relationship between osteocyte apoptosis and osteoclast-associated bone diseases such as osteoporosis and fragility fractures. The most prevalent notion refers to apoptotic osteocytes as the trigger of osteoclastogenesis and ensuing bone loss^36,52,71^. This is manifested by the fact that osteocyte apoptosis and ensuing resorption-related bone loss in response to fatigue loading^9^ and OVX^71^ were abolished by the application of a pan-caspase inhibitor. Accordingly, it has been established that the prevalence of apoptotic osteocytes is a causative determinant for enhanced osteoclastogenesis and ensuing bone loss^9,36,52,71,149–152^. Strikingly, multiple endocrine neoplasia type 1 (Men1) expression was significantly decreased in Men1^Runx2Cre^ mice, which was obtained by crossing Men1^tm1.1Zqw^ mice to Tg (Runx2-iCre) 1Jtuc mice. Meanwhile, it was exhibited by micro-computed tomography (Micro CT) that the obvious bone loss occurred in the distal femur of female Men1^Runx2Cre^ mice, resembling osteoporosis^153^, which implying Men1 has a crucial role in preserving bone integrity and suppressing the development of osteoporosis. As described above, apoptotic osteocytes in inflammatory conditions were found to elevate the secretion of the pro-osteoclastogenic and pro-inflammatory biomarkers such as TNF-α, IL-6, IL-11, RANKL, HMGB1^112,150^, as well as intercellular cell adhesion molecule-1 (ICAM-1)^152^, VEGF-A, IGF-1^93^, and DMP-1^154^, all of which have a crucial role in bone destruction and resorption (Fig. 5). Currently, accumulated evidence indicate that osteocyte apoptosis induced under pathologies (e.g., unloading/disuse, aging) initiates the increased expression level of NF-κB, thereby triggering RANK-induced osteoclastogenesis and bone resorption^52,99^. Aging-induced osteocytes apoptosis was demonstrated to promote bone loss through RANKL activation and sclerostin upregulation due to increased mitochondrial uncoupling and superoxide production (e.g., ROS)^46^. Based on above these findings, it is indicated that there exists an intrinsic and complex cross-talk mechanism between apoptotic osteocytes and osteoclastogenesis, while it is not fully deciphered and is still debatable. Of note, it was found that apoptotic osteocytes could serve not only as a direct role but also as an indirect role (i.g., by signaling to neighbor vital osteocytes) in regulating the migration and differentiation of osteoclasts precursor^155^ (Fig. 5). Indeed, it has been shown in an in vitro study that once mixed with osteocyte apoptotic bodies (OABs), mononuclear osteoclast precursors (OPs) could give rise to the preserving of OPs amounts and elevated the number and actions of TRACP^+^ cells. In contrast, whether in vivo or in vitro, apoptotic bodies (ABs) derived from osteoblasts exhibited no activity of osteoclastogenesis^150^. Notably, deletion of RANKL in osteocytes of Tnfsf11^flox/∆^ mice crossed with Dmp1-Cre mice (Tnfsf11^flox/∆^; Dmp1-Cre) diminished the activity of osteocytes in triggering the osteoclastogenesis, whereas it had no effects on osteoblasts, which indicates that RANKL derived from osteocyte might have an important role in triggering the osteoclastogenesis in vivo^156^. This is in consistent with the findings that HMGB1, released during osteocyte apoptosis, directly triggers osteoclastogenesis via activation of RAGE, and consequently increases pro-osteoclastogenic cytokine release (e.g., RANKL) from apoptotic osteocytes^93^. Until recently, it has been demonstrated that while the differentiation and formation of osteoclasts were not inhibited by Cx43^def^ CM with immunodepletion of HMGB1, it yet was suppressed by Cx43^def^ CM treated with an HMGB1 neutralizing antibody and then followed by immunodepletion of HMGB1, which indicates that the direct role of HMGB1, but not its indirect role, enhances apoptotic osteocytes-triggered osteoclastogenesis^157^. Similarly, Cx43^def^ osteocytic cells are found to undergo accelerated apoptosis and ensuing increased release of HMGB1, which directly stimulates pro-osteoclastogenic signal release from Cx43^def^ osteocytes^41^. Of interest, while Cx43^def^ osteocytes gave rise to the elevated number of empty lacunae, this situation was only positioned in sits of the cortical skeleton with dying osteocytes, implying that signaling biomarkers from dying or apoptotic osteocytes, but not that from healthy or vital osteocytes, are necessary to promote the differentiation and formation of osteoclasts^41^. Inconsistent with this notion, some evidence suggest that apoptotic osteocytes do not directly initiate osteoclastogenesis, but indirectly trigger that via the neighboring and viable osteocytes, as evidenced by the fact that removal of Cx43 in Cx43^∆Ot^ mice, compared with Cx43^fl/fl^ mice, significantly elevated the number of apoptotic osteocytes concomitant with targeted recruitment of osteoclasts, however, disconnection of mechanical signals transduction between the dying or apoptotic osteocytes (Cx43^def^) and the nearby healthy osteocytes could give rise to the increased expression levels of pro-osteoclastogenic biomarkers (e.g., RANKL) in vital osteocytes^158^. Mechanically, the expression level of RANKL was reported to be increased in viable and neighboring osteocytes upon osteocyte apoptosis, which, in turn, stimulates the differentiation and formation of osteoclasts, as well as subsequent focal bone resorption^52,159^. In this line, a report by Cheung WY et al.^160^ has shown that apoptotic osteocytes could activate the P2X7-receptor and pannexin-1 (Panx1) in skeleton upon fatigue to stimulate the production of RANKL in nearby vital osteocytes. Meanwhile, it was emphasized that ATP could is a pivotal mediator in regulating the process as described above. In addition, it was demonstrated that while the process of skeleton remodeling was stimulated by apoptotic osteocytes upon microdamage, the pro-osteoclastogenic and pro-inflammatory biomarkers (e.g., TNF-α, IL-6, IL-11, RANKL, HMGB1, etc) were mainly expressed in the nearby healthy osteocytes. Of interest, both apoptotic osteocytes and neighboring vital osteocytes in response to microdamage are positioned in a temporally and spatially determined model, which is in the line with the natural feature of targeted skeleton remodeling under the environment of microdamage^4^. Whether initiated by directly or indirectly, or even both, osteoclastogenesis-triggered bone resorption and bone loss due to unloading/disuse or sex steroids deficiency have been shown to, at least in part, be suppressed pharmacologically^1,149^. Intriguingly, it is also proposed that suppression of osteocyte apoptosis eliminated the expression level of RANKL derived from osteocytes, whereas, it did not prevent the resorption-related bone loss triggered by unloading/disuse, implying osteocyte apoptosis is independent of osteoclastogenesis-associated bone resorption process^161^. It also is evidenced by the result from osteocytic response to particles that inhibition of Caspase-3 induced a pro-osteoclastogenic effect through upregulation of the RANKL/OPG ratio, meanwhile, it also evoked the osteocytic osteolysis with the elevation of both carbonicanhydrase 2 (CA2) and cathepsin K (CTSK) expression^162^. This is likely due to different apoptotic mechanism upon various pathological agents. Until recently, it was reported that the expression and activity of matrix metalloproteinase 14 (MMP14) are elevated in the transgenic mice model (caPTH1R^Ot^), rather than in cKOPTH1R^Ot^ mice model (i.g., conditional knockout of PTH1R in osteocytes). As a result, activity of MMP14 increased the expression of soluble RANKL, which subsequently stimulated the differentiation of osteoclasts and ensuing bone resorption. Instead, while the activity of MMP14 was inhibited pharmacologically, the high level of bone remodeling and bone resorption was reduced in the caPTH1R^Ot^ mice model or mice exposed to chronic PTH elevation, which indicates that the resorption-associated bone loss seems to have no relations with osteocyte apoptosis^163^.

Here, it should be emphasized that apoptosis of osteoblasts was also shown to be directly induced under some pathological agents, such as hypoxia^164^, Es deficiency^165^, aging^166^, and GCs^167^, etc, which enhanced the reduction of the number of osteocyte processes, eventually leading to increased bone resorption and decreased BMD. However, autophagy or apoptosis of osteocytes, as the most abundant cells in bone, could have a more important role in osteoclastogenesis-triggered bone loss, compared with apoptosis of osteoblasts. Given the above, it is required to further investigate the exact role of osteocyte apoptosis, hopefully providing a more comprehensive explanation for various osteoclast-associated bone disorders.

## Conclusions and future perspectives

Indifferent from apoptosis, ferroptosis is not implicated in the activation of caspases, but depends on iron to enhance lipid peroxidation and initiate cell death. Indeed, it was suggested that the injury in bone marrow stromal cells resulted from chemotherapy could be regulated by ferroptosis, which offers an amenable target to decrease the side effects of ferroptosis initiaters^168^. In addition, it has also been demonstrated both in human and murine model that pyroptosis, a novel programmed cell death, has a pivotal role in inflammatory-related bone diseases with bone loss, such as osteomyelitis^169^ and diabetes mellitus-associated periodontitis^170^, which may be due to inhibition of proliferation and differentiation of osteoblasts. Nevertheless, whether the mechanism of ferroptosis or pyroptosis could occur in osteocytes remains ill-defined.

Currently, clinical anabolic drugs, such as teriparatide (PTH1-34)^171^, tetracycline^172^, and bisphosphonate^173^, etc, are illustrated to contribute to enhance bone formation. Nevertheless, both these drugs produce inevitable side effects due to lack of target-cell specificity of delivery systems. With a surge of material science and nanomedicine, several specific osteoblast-targeting delivery systems, such as aptamer-functionalized lipid nanoparticles (LNPs)^174^ and Ser-Asp-Ser-Ser-Asp-polyurethane (SDSSD-PU)^175^, have been updated from a tissue level to a cellular level. Though these delivery systems were shown to enhance the bone microarchitecture and elevate the bone mass in vitro, their safety and effectiveness in vivo remain a challenging issue. Most importantly, because of short of insight into mechanisms of therapy, the subsequent translational practice is also hindered. Of note, while osteocytes are indicated to master the process of bone metabolism by theirs interplay with osteoblasts and osteoclasts, osteocyte-targeting tools, as an anabolic strategy, have been rare. Until recently, it was reported by Qiao et al.^176^ that a PH-responsive osteocyte-targeting theranostic system, zoledronic acid (ZA)-anchored Gd-doped mesoporous silica-coated upconversion nanoparticles (UCMSs) loaded with plumbagin (PL) and polyacrylic acid (PAA), facilitated to accurately diagnose and effectively treat early bone metastasis of breast cancer. Thereby, targeted drugs that are transformed to only reach and act on osteocytes without affecting other bone tissue cells should also be highly desirable, as an anabolic strategy, to treat resorption-related bone diseases including osteoporosis and fragility fractures.

Taken together, vital osteocytes have been shown to be of paramount importance in preserving the mechanosensation and mechanoconduction of bone. Admittedly, some key anti-apoptotic biomarkers (e.g., Cx43, PGE2 FGF7, CCL7) have a crucial role in protecting osteocytic LCN and promoting intercellular communication. However, in the context of homeostasis unbalance under various pathological agents, key pro-apoptotic biomarkers (e.g., ROS, BNIP3, BAX, DMP1) could master the process of osteocytic apoptosis via activating intrinsic and complex signaling pathways. Once a bulk of apoptotic osteocytes occur, osteoclastogenesis may be initiated, consequently triggering and promoting the increased bone loss and bone fragility.

In addition, the lack of understanding about the actions of osteocytes cells death in human bone pathologies also constrains the attractive and proper actions of pharmacotherapeutics of targeting apoptosis. Accordingly, the potential cross-talk mechanisms whereby osteocyte apoptosis occurs in various states of pathologies and subsequently initiates osteoclastogenesis need to be more clearly established in future preclinical models for translation to human biology. Of course, it is important that other potential mechanisms should also be considered as osteocyte apoptosis and ensuing osteoclastogenesis, hopefully resulting in a more complete explanation for bone disorders under key pathological conditions.

## References

[1] Plotkin LI (2014). Apoptotic osteocytes and the control of targeted bone resorption. Curr. Osteoporos. Rep..

[2] Tsujimoto Y, Shimizu S (2015). Another way to die: autophagic programmed cell death. Cell Death Differ..

[3] Farr JN (2017). Targeting cellular senescence prevents age-related bone loss in mice. Nat. Med..

[4] Kennedy OD (2012). Activation of resorption in fatigue-loaded bone involves both apoptosis and active pro-osteoclastogenic signaling by distinct osteocyte populations. Bone.

[5] Aguirre JI (2006). Osteocyte apoptosis is induced by weightlessness in mice and precedes osteoclast recruitment and bone loss. J. Bone Miner. Res..

[6] Kogianni G (2004). Fas/CD95 is associated with glucocorticoid-induced osteocyte apoptosis. Life Sci..

[7] Lirani-Galvao AP (2009). Low-intensity electrical stimulation counteracts the effects of ovariectomy on bone tissue of rats: effects on bone microarchitecture, viability of osteocytes, and nitric oxide expression. Calcif. Tissue Int..

[8] Morita M (2017). Elevation of pro-inflammatory cytokine levels following anti-resorptive drug treatment is required for osteonecrosis development in infectious osteomyelitis. Sci. Rep..

[9] Tatsumi S (2007). Targeted ablation of osteocytes induces osteoporosis with defective mechanotransduction. Cell Metab..

[10] Bonewald LF (2011). The amazing osteocyte. J. Bone Miner. Res..

[11] Klein-Nulend J, Bakker AD, Bacabac G, Vatsa A, Weinbaum S (2013). Mechanosensation and transduction in osteocytes. Bone.

[12] Deepak V, Kayastha P, McNamara LM (2017). Estrogen deficiency attenuates fluid flow-induced [Ca(2+)]i oscillations and mechanoresponsiveness of MLO-Y4 osteocytes. FASEB J..

[13] van Hove RP (2009). Osteocyte morphology in human tibiae of different bone pathologies with different bone mineral density — Is there a role for mechanosensing?. Bone.

[14] Milovanovic P (2013). Osteocytic canalicular networks: morphological implications for altered mechanosensitivity. ACS Nano..

[15] Qiu S, Rao DS, Fyhrie DP, Palnitkar S, Parfitt AM (2005). The morphological association between microcracks and osteocyte lacunae in human cortical bone. Bone.

[16] Busse B (2010). Decrease in the osteocyte lacunar density accompanied by hypermineralized lacunar occlusion reveals failure and delay of remodeling in aged human bone. Aging Cell..

[17] Fimia GM, Piacentini M (2010). Regulation of autophagy in mammals and its interplay with apoptosis. Cell. Mol. Life. Sci..

[18] Mukhopadhyay S, Panda PK, Sinha N, Das DN, Bhutia SK (2014). Autophagy and apoptosis: where do they meet?. Apoptosis.

[19] Marino G, Niso-Santano M, Baehrecke EH, Kroemer G (2014). Self-consumption: the interplay of autophagy and apoptosis. Nat. Rev. Mol. Cel. Biol..

[20] Mizushima N, Levine B, Cuervo AM, Klionsky DJ (2008). Autophagy fights disease through cellular self-digestion. Nature.

[21] Piemontese M (2016). Low bone mass and changes in the osteocyte network in mice lacking autophagy in the osteoblast lineage. Sci. Rep..

[22] Zahm AM, Bohensky J, Adams CS, Shapiro IM, Srinivas V (2011). Bone cell autophagy is regulated by environmental factors. Cells Tissues Organs.

[23] Yao W, Dai W, Jiang JX, Lane NE (2013). Glucocorticoids and osteocyte autophagy. Bone.

[24] Filomeni G, De Zio D, Cecconi F (2015). Oxidative stress and autophagy: the clash between damage and metabolic needs. Cell Death Differ..

[25] Maiuri MC, Kroemer G (2015). Autophagy in stress and disease. Cell Death Differ..

[26] Monastyrska I, Rieter E, Klionsky DJ, Reggiori F (2009). Multiple roles of the cytoskeleton in autophagy. Biol. Rev. Camb. Philos. Soc..

[27] Zhu Y (2010). Beclin 1 cleavage by caspase-3 inactivates autophagy and promotes apoptosis. Protein Cell..

[28] Florencio-Silva R, Sasso GRS, Sasso-Cerric E, Simõesa MJ, Cerricy PS (2018). Effects of estrogen status in osteocyte autophagy and its relation to osteocyte viability in alveolar process of ovariectomized rats. Biomed. Pharmacother..

[29] Wang K (2019). A novel osteogenic cell line that differentiates into GFP-tagged osteocytes and forms mineral with a bone-like lacunocanalicular structure. J. Bone Miner. Res..

[30] Bernhardt A, Wolf S, Weiser E, Vater C, Gelinsky M (2020). An improved method to isolate primary human osteocytes from bone. Biomed. Technol..

[31] Hemmatian H, Bakker AD, Klein-Nulend J, van Lenthe GH (2017). Aging, osteocytes, and mechanotransduction. Cur. Osteoporos. Rep..

[32] Galea GL (2017). Old age and the associated impairment of bones’ adaptation to loading are associated with transcriptomic changes in cellular metabolism, cell-matrix interactions and the cell cycle. Gene.

[33] Nishida T, Kubota S, Yokoi H, Mukoyama M, Takigawa M (2019). Roles of matricellular CCN2 deposited by osteocytes in osteoclastogenesis and osteoblast differentiation. Sci. Rep..

[34] Bach-Gansmo FL (2016). Osteocyte lacunar properties and cortical microstructure in human iliac crest as a function of age and sex. Bone.

[35] Lai X (2015). The dependences of osteocyte network on bone compartment, age, and disease. Bone Res..

[36] Jilka RL, O’Brien CA (2016). The role of osteocytes in age-related bone loss. Curr. Osteoporos. Rep..

[37] Plotkin LI, Bellido T (2016). Osteocytic signalling pathways as therapeutic targets for bone fragility. Nat. Rev. Endocrinol..

[38] Noble BS (2003). Mechanical loading: biphasic osteocyte survival and targeting of osteoclasts for bone destruction in rat cortical bone. Am. J. Physiol. Cell. Physiol..

[39] Javaheri B, Pitsillides AA (2019). Aging and mechanoadaptive responsiveness of bone. Curr. Osteoporos. Rep..

[40] Chastin SF, Mandrichenko O, Helbostadt JL, Skelton DA (2014). Associations between objectively- measured sedentary behaviour and physical activity with bone mineral density in adults and older adults, the NHANES study. Bone.

[41] Davis HM (2017). Disruption of the Cx43/miR21 pathway leads to osteocyte apoptosis and increased osteoclastogenesis with aging. Aging Cell.

[42] Talbot J (2018). Connexin43 intercellular communication drives the early differentiation of human bone marrow stromal cells into osteoblasts. J. Cell Physiol..

[43] Xu H (2015). Connexin 43 channels are essential for normal bone structure and osteocyte viability. J. Bone Miner. Res..

[44] Joiner DM, Tayim RJ, McElderry JD, Morris MD, Goldstein SA (2014). Aged male rats regenerate cortical bone with reduced osteocyte density and reduced secretion of nitric oxide after mechanical stimulation. Calcif. Tissue Int..

[45] Figueiredo PA, Powers SK, Ferreira RM, Appell HJ, Duarte JA (2009). Aging impairs skeletal muscle mitochondrial bioenergetic function. J. Gerontol. A Biol. Sci. Med. Sci..

[46] Kang C, Chung E, Diffee G, Ji LL (2013). Exercise training attenuates aging-associated mitochondrial dysfunction in rat skeletal muscle: role of PGC-1alpha. Exp. Gerontol..

[47] Kobayashi K (2015). Mitochondrial superoxide in osteocytes perturbs canalicular networks in the setting of age-related osteoporosis. Sci. Rep..

[48] Almeida M (2007). Skeletal involution by age-associated oxidative stress and its acceleration by loss of sex steroids. J. Biol. Chem..

[49] Korotchkina LG (2010). The choice between p53-induced senescence and quiescence is determined in part by the mTOR pathway. Aging.

[50] Lloyd SA (2014). Interdependence of muscle atrophy and bone loss induced by mechanical unloading. J. Bone Miner. Res..

[51] Mann V, Huber C, Kogianni G, Jones D, Noble B (2006). The influence of mechanical stimulation on osteocyte apoptosis and bone viability in human trabecular bone. J. Musculoskelet. Neuronal. Interact..

[52] Cabahug-Zuckerman P (2016). Osteocyte apoptosis caused by hindlimb unloading is required to trigger osteocyte RANKL production and subsequent resorption of cortical and trabecular bone in mice femurs. J. Bone Miner. Res..

[53] Lin C (2009). Sclerostin mediates bone response to mechanical unloading through antagonizing Wnt/beta-catenin signaling. J. Bone Miner. Res..

[54] Tan SD, Bakker AD, Semeins CM, Kuijpers-Jagtman AM, Klein-Nulend J (2008). Inhibition of osteocyte apoptosis by fluid flow is mediated by nitric oxide. Biochem. Biophys. Res. Commun..

[55] Plotkin LI (2005). Mechanical stimulation prevents osteocyte apoptosis: requirement of integrins, Src kinases, and ERKs. Am. J. Physiol. Cell. Physiol..

[56] Kitase Y (2010). Mechanical induction of PGE2 in osteocytes blocks glucocorticoid-induced apoptosis through both the beta-catenin and PKA pathways. J. Bone Miner. Res..

[57] Storlino G (2020). Irisin prevents disuse-induced osteocyte apoptosis. J. Bone Miner. Res..

[58] Kim H (2019). Irisin mediates effects on bone and fat via alphaV integrin receptors. Cell.

[59] Liu XY (2018). FGF-7 dictates osteocyte cell processes through Beta-catenin transduction. Sci. Rep..

[60] Hinoi E (2012). Positive regulation of osteoclastic differentiation by growth differentiation factor 15 upregulated in osteocytic cells under hypoxia. J. Bone Miner. Res..

[61] van Breukelen F, Krumschnabel G, Podrabsky JE (2010). Vertebrate cell death in energy-limited conditions and how to avoid it: what we might learn from mammalian hibernators and other stress- tolerant vertebrates. Apoptosis.

[62] Montesi M (2016). Hypoxia mediates osteocyte ORP150 expression and cell death in vitro. Mol. Med. Rep..

[63] Guo D (2010). Identification of osteocyte-selective proteins. Proteomics.

[64] Burr DB (2019). Stress concentrations and bone microdamage: John Currey’s contributions to understanding the initiation and arrest of cracks in bone. Bone.

[65] Liu X (2019). Spatiotemporal distribution of linear microcracks and diffuse microdamage following daily bouts of fatigue loading of rat ulnae. J. Orthop. Res..

[66] Verborgt O, Tatton NA, Majeska RJ, Schaffler MB (2002). Spatial distribution of Bax and Bcl-2 in osteocytes after bone fatigue: complementary roles in bone remodeling regulation?. J. Bone Miner. Res..

[67] Hagan ML (2020). Decreased pericellular matrix production and selection for enhanced cell membrane repair may impair osteocyte responses to mechanical loading in the aging skeleton. Aging Cell..

[68] Hagan ML (2019). Inhibition of osteocyte membrane repair activity via dietary Vitamin E deprivation impairs osteocyte survival. Calcif. Tissue Int..

[69] Hoshi K (2014). Compressive force-produced CCN2 induces osteocyte apoptosis through ERK1/2 pathway. J. Bone Miner. Res..

[70] Almeida M (2017). Estrogens and androgens in skeletal physiology and pathophysiology. Physiol. Rev..

[71] Emerton KB (2010). Osteocyte apoptosis and control of bone resorption following ovariectomy in mice. Bone.

[72] Almeida M, Han L, Ambrogini E, Bartell SM, Manolagas SC (2010). Oxidative stress stimulates apoptosis and activates NF-kappaB in osteoblastic cells via a PKCbeta/p66shc signaling cascade: counter regulation by estrogens or androgens. Mol. Endocrinol..

[73] Fu J (2018). miR-199a-3p is involved in estrogen-mediated autophagy through the IGF-1/mTOR pathway in osteocyte-like MLO-Y4 cells. J. Cell Physiol..

[74] Qi M (2017). Autophagy maintains the function of bone marrow mesenchymal stem cells to prevent estrogen deficiency-induced osteoporosis. Theranostics.

[75] Wei Y, Huang J (2019). Role of estrogen and its receptors mediated-autophagy in cell fate and human diseases. J. Steroid Biochem. Mol. Biol..

[76] de Souza Faloni AP (2011). Jaw and long bone marrows have a different osteoclastogenic potential. Calcif. Tissue Int..

[77] Hayashi M (2019). Autoregulation of osteocyte sema3A orchestrates estrogen action and counteracts bone aging. Cell Metab..

[78] Wang T, Yu X, He C (2019). Pro-inflammatory cytokines: cellular and molecular drug targets for glucocorticoid-induced-osteoporosis via osteocyte. Curr. Drug. Targets.

[79] Fowler TW (2017). Glucocorticoid suppression of osteocyte perilacunar remodeling is associated with subchondral bone degeneration in osteonecrosis. Sci. Rep..

[80] Shen G (2018). Autophagy as a target for glucocorticoid-induced osteoporosis therapy. Cell. Mol. Life. Sci..

[81] Jia J (2011). Glucocorticoid dose determines osteocyte cell fate. FASEB J..

[82] Plotkin LI, Manolagas SC, Bellido T (2007). Glucocorticoids induce osteocyte apoptosis by blocking focal adhesion kinase-mediated survival: evidence for inside-out signaling leading to anoikis. J. Biol. Chem..

[83] Kitase Y (2014). CCL7 is a protective factor secreted by mechanically loaded osteocytes. J. Dent. Res..

[84] Zhu L (2017). Parathyroid hormone (PTH) induces autophagy to protect osteocyte cell survival from dexamethasone damage. Med. Sci. Monit..

[85] Jia YB (2017). Inhibitory effects of vitamin E on osteocyte apoptosis and DNA oxidative damage in bone marrow hemopoietic cells at early stage of steroid-induced femoral head necrosis. Mol. Med. Rep..

[86] Agrawal M (2011). Bone, inflammation, and inflammatory bowel disease. Cur. Osteoporos. Rep..

[87] Abrahami D (2018). Dipeptidyl peptidase-4 inhibitors and incidence of inflammatory bowel disease among patients with type 2 diabetes: population based cohort study. BMJ.

[88] Mazzaferro S (2018). Bone, inflammation and the bone marrow niche in chronic kidney disease: what do we know?. Nephrol. Dial. Transplant..

[89] Allison DJ, Ditor DS (2015). Immune dysfunction and chronic inflammation following spinal cord injury. Spinal Cord..

[90] Metzger CE, Narayanan A, Zawieja DC, Bloomfield SA (2017). Inflammatory bowel disease in a rodent model alters osteocyte protein levels controlling bone turnover. J. Bone Miner. Res..

[91] Narayanan SA, Metzger CE, Bloomfield SA, Zawieja DC (2018). Inflammation induced lymphatic rchitecture and bone turnover changes are ameliorated by irisin treatment in chronic inflammatory bowel disease. FASEB J..

[92] Ahuja SS (2003). CD40 ligand blocks apoptosis induced by tumor necrosis factor-alpha, glucocorticoids, and etoposide in osteoblasts and the osteocyte-like cell line murine long bone osteocyte-Y4. Endocrinology.

[93] Chen H (2017). Advanced glycation end products induced IL-6 and VEGF-A production and apoptosis in osteocyte-like MLO-Y4 cells by activating RAGE and ERK1/2, P38 and STAT3 signalling pathways. Int. Immunopharmacol..

[94] Zhang C (2019). FOXO1 mediates advanced glycation end products induced mouse osteocyte-like MLO- Y4 cell apoptosis and dysfunctions. J. Diabetes Res..

[95] Li JKarim MA, Che H, Geng Q, Miao D (2020). Deletion of p16 prevents estrogen deficiency-induced osteoporosis by inhibiting oxidative stress and osteocyte senescence. Am. J. Transl. Res..

[96] Takeno A (2015). Activation of AMP-activated protein kinase protects against homocysteine-induced apoptosis of osteocytic MLO-Y4 cells by regulating the expressions of NADPH oxidase 1 (Nox1 and Nox2). Bone.

[97] Brown DI, Griendling KK (2009). Nox proteins in signal transduction. Free Radic. Biol. Med..

[98] Farr JN (2016). Identification of senescent cells in the bone microenvironment. J. Bone Miner. Res..

[99] Piemontese M (2016). Old age causes de novo intracortical bone remodeling and porosity in mice. Jci. Insight.

[100] Zhai YK (2014). Icariin stimulates the osteogenic differentiation of rat bone marrow stromal cells via activating the PI3K-AKT-eNOS-NO-cGMP-PKG. Bone.

[101] Khan MP (2015). Pathophysiological mechanism of bone Loss in type 2 diabetes involves inverse regulation of osteoblast function by PGC-1alpha and skeletal muscle atrogenes: adipoR1 as a potential target for reversing diabetes-induced osteopenia. Diabetes.

[102] Baldelli S, Aquilano K, Ciriolo MR (2014). PGC-1alpha buffers ROS-mediated removal of mitochondria during myogenesis. Cell. Death. Dis..

[103] Son M (2017). Age dependent accumulation patterns of advanced glycation end product receptor (RAGE) ligands and binding intensities between RAGE and its ligands differ in the liver, kidney, and skeletal muscle. Immun. Ageing.

[104] Sorci G, Riuzzi F, Giambanco I, Donato R (2013). RAGE in tissue homeostasis, repair and regeneration. Biochim. Biophys. Acta.

[105] Cheng YZ (2018). Irbesartan attenuates advanced glycation end products-mediated damage in diabetes- associated osteoporosis through the AGEs/RAGE pathway. Life. Sci..

[106] Ramasamy R, Yan SF, Schmidt AM (2012). Advanced glycation endproducts: from precursors to RAGE: round and round we go. Amino. Acids.

[107] Tanaka K, Yamaguchi T, Kanazawa I, Sugimoto T (2015). Effects of high glucose and advanced glycation end products on the expressions of sclerostin and RANKL as well as apoptosis in osteocyte- like MLO-Y4-A2 cells. Biochem. Biophys. Res. Commun..

[108] Notsu M (2017). Advanced glycation end products (AGEs) increases apoptosis and the expression of sclerostin by stimulating TGF-beta expression and secretion in osteocyte-like MLO-Y4-A2 cells. Calcif. Tissue Int..

[109] Yu Y, Tang D, Kang R (2015). Oxidative stress-mediated HMGB1 biology. Front. Physiol..

[110] Feng L (2016). HMGB1 promotes the secretion of multiple cytokines and potentiates the osteogenic differentiation of mesenchymal stem cells through the Ras/MAPK signaling pathway. Exp. Ther. Med..

[111] Yang H, Wang H, Andersson U (2020). Targeting inflammation driven by HMGB1. Front. Immunol..

[112] Zhou Z (2008). HMGB1 regulates RANKL-induced osteoclastogenesis in a manner dependent on RAGE. J. Bone Miner. Res..

[113] Yoshida T, Flegler A, Kozlov A, Stern PH (2009). Direct inhibitory and indirect stimulatory effects of RAGE ligand S100 on sRANKLinduced osteoclastogenesis. J. Cell. Biochem..

[114] Davalos AR (2013). p53-dependent release of Alarmin HMGB1 is a central mediator of senescent phenotypes. J. Cell. Biol..

[115] Pesce Viglietti AI (2016). Brucella abortus invasion of osteocytes modulates vonnexin 43 and integrin expression and induces osteoclastogenesis via receptor activator of NF-kappaB ligand and tumor necrosis factor alpha secretion. Infect. Immun..

[116] Yang R (2016). Autophagy plays a protective role in tumor necrosis factor-alpha-induced apoptosis of bone marrow-derived mesenchymal stem cells. Stem. Cells Dev..

[117] Schmidt M (2001). Role of the CD95/CD95 ligand system in glucocorticoid-induced monocyte apoptosis. J. Immunol..

[118] Wang L (2015). Osteoblast-induced osteoclast apoptosis by FAS ligand/FAS pathway is required for maintenance of bone mass. Cell Death Differ..

[119] Boya P, Reggiori F, Codogno P (2013). Emerging regulation and functions of autophagy. Nat. Cell. Biol..

[120] Fimia GM, Kroemer G, Piacentini M (2013). Molecular mechanisms of selective autophagy. Cell. Death. Differ..

[121] Shapiro IM, Layfield R, Lotz M, Settembre C, Whitehouse C (2014). Boning up on autophagy: the role of autophagy in skeletal biology. Autophagy.

[122] Yurube T, Ito M, Kakiuchi Y, Kuroda R, Kakutani K (2020). Autophagy and mTOR signaling during intervertebral disc aging and degeneration. Jor. Spine.

[123] Onal M (2013). Suppression of autophagy in osteocytes mimics skeletal aging. J. Biol. Chem..

[124] Chen K, Yang YH, Jiang SD, Jiang LS (2014). Decreased activity of osteocyte autophagy with aging may contribute to the bone loss in senile population. Histochem. Cell. Biol..

[125] Greenhill C (2016). Bone: autophagy regulates bone growth in mice. Nat. Rev. Endocrinol..

[126] Wang T, Liu X, He C (2020). Glucocorticoid-induced autophagy and apoptosis in bone. Apoptosis.

[127] Djavaheri-Mergny M, Maiuri MC, Kroemer G (2010). Cross talk between apoptosis and autophagy by caspase-mediated cleavage of Beclin 1. Oncogene.

[128] Powell WF (2011). Targeted ablation of the PTH/PTHrP receptor in osteocytes impairs bone structure and homeostatic calcemic responses. J. Endocrinol..

[129] Ansari N (2018). Autocrine and paracrine regulation of the murine skeleton by osteocyte-derived parathyroid hormone-related protein. J. Bone Miner. Res..

[130] Chandra A (2014). PTH1-34 alleviates radiotherapy-induced local bone loss by improving osteoblast and osteocyte survival. Bone.

[131] Sugiyama T (2008). Mechanical loading enhances the anabolic effects of intermittent parathyroid hormone (1-34) on trabecular and cortical bone in mice. Bone.

[132] Shibamoto A (2018). Effect of high-frequency loading and parathyroid hormone administration on peri-implant bone healing and osseointegration. Int. J. Oral. Sci..

[133] Maycas M (2015). Role of the parathyroid hormone type 1 receptor (PTH1R) as a mechanosensor in osteocyte survival. J. Bone Miner. Res..

[134] Bellido T, Saini V, Pajevic PD (2013). Effects of PTH on osteocyte function. Bone.

[135] Yavropoulou MP, Michopoulos A, Yovos JG (2017). PTH and PTHR1 in osteocytes. New insights into old partners. Hormones.

[136] Liedert A, Kaspar D, Blakytny R, Claes L, Ignatius A (2006). Signal transduction pathways involved in mechanotransduction in bone cells. Biochem. Biophys. Res. Commun..

[137] Rhee Y (2011). PTH Receptor Signaling in Osteocytes governs periosteal bone formation and intracortical remodeling. J. Bone Miner. Res..

[138] Bivi N, Lezcano V, Romanello M, Bellido T, Plotkin LI (2011). Connexin43 interacts with barrestin: a prerequisite for osteoblast survival induced by parathyroid hormone. J. Cell. Biochem..

[139] Ugland H, Naderi S, Brech A, Collas P, Blomhoff HK (2011). cAMP induces autophagy via a novel pathway involving ERK, cyclin E and Beclin 1. Autophagy.

[140] de Castro LF, Maycas M, Bravo B, Esbrit P, Gortazar A (2015). VEGF receptor 2 (VEGFR2) activation is essential for osteocyte survival induced by mechanotransduction. J. Cell. Physiol..

[141] Clarkin CE, Gerstenfeld LC (2013). VEGF and bone cell signalling: an essential vessel for communication?. Cell. Biochem. Funct..

[142] Domazetovic V (2017). Entroges inhibits starvation-induced apoptosis in osteocytes by a redox-independent process involving association of JNK and glutathione S-transferase P-1. FEBS Open. Bio..

[143] Plotkin LI, Bivi N, Bellido T (2011). A bisphosphonate that does not affect osteoclasts prevents osteoblast and osteocyte apoptosis and the loss of bone strength induced by glucocorticoids in mice. Bone.

[144] Gortazar AR, Martin-Millan M, Bravo B, Plotkin LI, Bellido T (2013). Crosstalk between caveolin-1/extracellular signal-regulated kinase (ERK) and beta-catenin survival pathways in osteocyte mechanotransduction. J. Biol. Chem..

[145] Kuang MJ (2019). Exosomes derived from Wharton’s jelly of human umbilical cord mesenchymal stem cells reduce osteocyte apoptosis in glucocorticoid-induced osteonecrosis of the femoral head in rats via the miR-21-PTEN-AKT signalling pathway. Int. J. Biol. Sci..

[146] Hayashi M (2012). Osteoprotection by semaphorin 3A. Nature.

[147] Hwang JY (2006). Association of PLXNA2 polymorphisms with vertebral fracture risk and bone mineral density in postmenopausal Korean population. Osteoporos. Int..

[148] Marathe N, Rangaswami H, Zhuang S, Boss GR, Pilz RB (2012). Pro-survival effects of 17beta- estradiol on osteocytes are mediated by nitricoxide/cGMP via differential actions of cGMP-dependent protein kinases I and II. J. Biol. Chem..

[149] Chai S, Wan L, Wang JL, Huang JC, Huang HX (2019). Gushukang inhibits osteocyte apoptosis and enhances BMP-2/Smads signaling pathway in ovariectomized rats. Phytomedicine.

[150] Kogianni G, Mann V, Noble BS (2008). Apoptotic bodies convey activity capable of initiating osteoclastogenesis and localized bone destruction. J. Bone Miner. Res..

[151] Cardoso L (2009). Osteocyte apoptosis controls activation of intracortical resorption in response to bone fatigue. J. Bone Miner. Res..

[152] Cheung WY, Simmons CA, You L (2012). Osteocyte apoptosis regulates osteoclast precursor adhesion via osteocytic IL-6 secretion and endothelial ICAM-1 expression. Bone.

[153] Liu P (2017). Loss of menin in osteoblast lineage affects osteocyte-osteoclast crosstalk causing osteoporosis. Cell Death Differ..

[154] He F (2019). Irradiation-induced osteocyte damage promotes HMGB1-mediated osteoclastogenesis in vitro. J. Cell Physiol..

[155] Al-Dujaili SA (2011). Apoptotic osteocytes regulate osteoclast precursor recruitment and differentiation in vitro. J. Cell. Biochem..

[156] Nakashima T (2011). Evidence for osteocyte regulation of bone homeostasis through RANKL expression. Nat. Med..

[157] Davis HM (2019). High mobility group box 1 protein regulates osteoclastogenesis through direct actions on osteocytes and osteoclasts in vitro. J. Cell. Biochem..

[158] Bivi N (2012). Cell autonomous requirement of connexin 43 for osteocyte survival: consequences for endocortical resorption and periosteal bone formation. J. Bone Miner. Res..

[159] Kennedy OD, Laudier DM, Majeskam RJ, Sun HB, Schaffler MB (2014). Osteocyte apoptosis is required for production of osteoclastogenic signals following bone fatigue in vivo. Bone.

[160] Cheung WY (2016). Pannexin-1 and p2X7-receptor are required for apoptotic osteocytes in fatigued bone to trigger RANKL production in neighboring bystander osteocytes. J. Bone Miner. Res..

[161] Plotkin LI (2015). Inhibition of osteocyte apoptosis prevents the increase in osteocytic receptor activator of nuclear factor kappaB ligand (RANKL) but it does not stop bone resorption or the loss of bone induced by unloading. J. Biol. Chem..

[162] Ormsby RT (2019). Osteocytes respond to particles of clinically-relevant conventional and cross-linked polyethylene and metal alloys by up-regulation of resorptive and inflammatory pathways. Acta Biomater..

[163] Delgado-Calle J (2018). MMP14 is a novel target of PTH signaling in osteocytes that controls resorption by regulating soluble RANKL production. Faseb. J..

[164] Yang CN (2019). Simvastatin alleviates bone resorption in apical periodontitis possibly by inhibition of mitophagy-related osteoblast apoptosis. Int. Endod. J..

[165] Zhang Y (2020). PSMC6 promotes osteoblast apoptosis through inhibiting PI3K/AKT signaling pathway activation in ovariectomy-induced osteoporosis mouse model. J. Cell Physiol..

[166] Moriishi T (2016). Overexpression of BCLXL in osteoblasts inhibits osteoblast apoptosis and increases bone volume and strength. J. Bone Miner. Res..

[167] Deng S (2019). Dexamethasone induces osteoblast apoptosis through ROS-PI3K/AKT/GSK3β signaling pathway. Biomed. Pharmacother..

[168] Song X (2016). FANCD2 protects against bone marrow injury from ferroptosis. Biochem. Biophys. Res. Commun..

[169] Zhu X (2019). Inhibition of pyroptosis attenuates staphylococcus aureus-induced bone injury in traumatic osteomyelitis. Ann. Transl. Med..

[170] Yang L, Liu J, Shan Q, Geng G, Shao P (2020). High glucose inhibits proliferation and differentiation of osteoblast in alveolar bone by inducing pyroptosis. Biochem. Biophys. Res. Commun..

[171] Daddona PE, Matriano JA, Mandema J, Maa YF (2011). Parathyroid hormone (1–34)-coated micro needle patch system: clinical pharmacokinetics and pharmacodynamics for treatment of osteoporosis. Pharm. Res..

[172] Neale JR (2009). Bone selective effect of an estradiol conjugate with a novel tetracycline-derived bone-targeting agent. Bioorg. Med. Chem. Lett..

[173] Hirabayashi H, Fujisaki J (2003). Bone-specific drug delivery systems. Clin. Pharmacokinet..

[174] Liang C (2015). Aptamer-functionalized lipid nanoparticles targeting osteoblasts as a novel RNA interference-based bone anabolic strategy. Nat. Med..

[175] Sun Y (2016). Osteoblast-targeting-peptide modified nanoparticle for siRNA/microRNA delivery. ACS Nano..

[176] Qiao H (2017). Targeting osteocytes to attenuate early breast cancer bone metastasis by theranostic upconversion nanoparticles with responsive plumbagin release. ACS Nano..

